# Circadian rhythm abnormalities and autonomic dysfunction in patients with Chronic Fatigue Syndrome/Myalgic Encephalomyelitis

**DOI:** 10.1371/journal.pone.0198106

**Published:** 2018-06-06

**Authors:** Trinitat Cambras, Jesús Castro-Marrero, Maria Cleofé Zaragoza, Antoni Díez-Noguera, José Alegre

**Affiliations:** 1 Chronobiology Group, Department of Physiology and Biochemistry, Faculty of Pharmacy and Food Sciences, Universitat de Barcelona, Barcelona, Spain; 2 CFS/ME Unit, Vall d’Hebron University Hospital Research Institute, Universitat Autònoma de Barcelona, Barcelona, Spain; 3 Clinical Research Department, Laboratorios Viñas, Barcelona, Spain; CNRS, University of Strasbourg, FRANCE

## Abstract

Chronic Fatigue Syndrome/Myalgic Encephalomyelitis (CFS/ME) patients frequently show autonomic symptoms which may be associated with a hypothalamic dysfunction. This study aimed to explore circadian rhythm patterns in rest and activity and distal skin temperature (DST) and their association with self-reported outcome measures, in CFS/ME patients and healthy controls at two different times of year. Ten women who met both the 1994 CDC/Fukuda definition and 2003 Canadian criteria for CFS/ME were included in the study, along with ten healthy controls matched for age, sex and body mass index. Self-reported measures were used to assess fatigue, sleep quality, anxiety and depression, autonomic function and health-related quality of life. The ActTrust actigraph was used to record activity, DST and light intensity, with data intervals of one minute over seven consecutive days. Sleep variables were obtained through actigraphic analysis and from subjective sleep diary. The circadian variables and the spectral analysis of the rhythms were calculated. Linear regression analysis was used to evaluate the relationship between the rhythmic variables and clinical features. Recordings were taken in the same subjects in winter and summer. Results showed no differences in rhythm stability, sleep latency or number of awakenings between groups as measured with the actigraph. However, daily activity, the relative amplitude and the stability of the activity rhythm were lower in CFS/ME patients than in controls. DST was sensitive to environmental temperature and showed lower nocturnal values in CFS/ME patients than controls only in winter. A spectral analysis showed no differences in phase or amplitude of the 24h rhythm, but the power of the second harmonic (12h), revealed differences between groups (controls showed a post-lunch dip in activity and peak in DST, while CFS/ME patients did not) and correlated with clinical features. These findings suggest that circadian regulation and skin vasodilator responses may play a role in CFS/ME.

## Introduction

Chronic Fatigue Syndrome, also referred to as Myalgic Encephalomyelitis (CFS/ME), is a debilitating chronic, multi-systemic neuroimmune condition, probably of multifactorial etiology. There are no clinically established diagnostic tests, nor are any FDA-approved drugs available for treatment. CFS/ME is estimated affects approximately 17 million children/adolescents and adults worldwide. It affects females more than males and causes high levels of work disability in adults or poor school performance in adolescents [1].

CFS/ME is characterized by severe disabling fatigue lasting more than six months that does not improve with rest, worsens with minimal physical and mental exertion and cannot be explained by any underlying medical condition. During the course of the illness, CFS/ME patients also experience relapses and partial remissions of the common features which include pathological dysregulation of the neuro-endocrine, immunological, gastrointestinal, autonomic systems with cellular energy metabolism/ion transport impairments of varying onset, duration, frequency and severity, reviewed in [1]. Symptoms can persist for years, and few patients regain their premorbid level of health or functioning [2]. The duration of CFS/ME and the potentially debilitating consequences of its symptoms significantly increase the economic burden and establish the condition as a top-level health problem in today’s society [3].

The aetiology of CFS/ME remains unclear. Although muscle function is altered in this condition [4–6] there is now a growing body of literature that associates autonomic dysfunction with hypothalamic-pituitary- adrenal axis impairment [7–9].

Moreover, sleep disturbances and insomnia are very common, with a pattern of restlessness and difficulty in falling and remaining sleep [10,11]. A high percentage of naps may also be observed, related to strategies for mitigating pain or compensating for the lack of sleep at night [12].

Circadian rhythms are prominent in the regulation of many physiological processes that are crucial to health [13,14]. Patients with CFS/ME may show evidence of circadian rhythm disturbances, but no clear results about this subject have yet been found. For example, Tomoda *et al* [15] found variations in the amplitude and phase of the rhythm of internal body temperature in children and adolescents, but other researchers found no differences in the rhythmic manifestations [16–18]. Other studies suggest a dissociation between body temperature rhythm and melatonin secretion rate in CFS/ME patients [19], although they do not conclude whether it is endogenous or due to the differences in patients’ lifestyles. All this suggests that circadian rhythms in these patients needs to be explored, especially since the balance between the sympathetic and parasympathetic nervous systems, indicative of autonomic function, is mainly regulated by the circadian control of the suprachiasmatic nuclei [20,21].

Actigraphy is a validated, non-invasive and objective tool for studying circadian patterns in natural conditions, in which individuals following their normal routine in their own family environment. The activity detected with the accelerometer correlates well with the parameters obtained from polysomnography [22,23], such as sleep efficiency and fragmentation. Actigraphy reports in CFS/ME objectively illustrate the reduction in activity, characteristic of the condition, with lower activity during the day in CFS/ME patients compared with healthy controls [24].

Apart from activity, the daily variation in the Distal Skin Temperature (DST) is also widely used to measure circadian rhythmicity. This variable presents a very robust rhythmic pattern with a circadian profile that is practically the reverse of the central temperature [25]. DST values increase during the night; they are low during the day, although they also rise during daytime sleep. In fact, sleep patterns are associated more with the rise in peripheral temperature than with the nocturnal lowering of the central temperature [26,27]. Since DST indirectly reflects the degree of skin vasodilatation, it might be used as an indicator of autonomic dysfunction, an important component into the pathophysiology of CFS/ME [28]. We hypothesized that altered circadian rhythm variables could contribute to the risk for CFS/ME.

Thus, the aims of present study are: 1) to evaluate possible disruptions of the autonomic function in a Spanish CFS/ME cohort by studying the circadian profiles of rest-activity, DST and light exposure; and 2) to determine whether changes in circadian rhythms correlate with worsening clinical symptoms in this cohort. Since environmental temperature could interfere with the results, we carried out the experiment in winter and summer.

## Materials and methods

### Participants

A prospective, cross-sectional case-control study of 10 consecutive women with CFS/ME and 10 non-fatigued healthy controls matched for age (mean age ± SEM, 48.7 ± 2.8 years), sex (all women) and BMI (mean BMI ± SEM, 25.07 ± 0.97 kg/m^2^) were recruited at a single outpatient tertiary-referral centre (CFS/ME Clinical Unit, Vall d’Hebron University Hospital, Barcelona, Spain) from January through March 2017. Patients were potentially eligible for the study if they fulfilled both the 1994 CDC/Fukuda definition [29] and 2003 Canadian criteria for CFS/ME [30]. A CFS/ME specialist diagnosed all patients who took part in the study and also conducted the clinical assessments of all participants. All study participants were of Caucasian descent and from the same geographical area (residents of Catalonia, Spain) at the time of study. Their sociodemographic data and clinical characteristics are shown in Table 1.

**Table 1:** Baseline demographic and clinical features of study participants.

Variable	Winter			Summer				
	CFS/ME	HC	p	CFS/ME	HC	p	p	
	n = 10	n = 10		n = 10	n = 8		season	group
**Age (years)** ^a^	50.6 ± 1.39	48.7 ± 4.13	0.704	--				--
**BMI (kg/m2)**^a^	26.3 ± 1.79	23.8 ± 0.66	0.222					
**FIS-40**								
**Global score (0–160)**	135.3 ± .46	31.8 ± 8.82	***0*.*000***	136.9 ± 5.03	28.5 ± 7.63	***0*.*000***	0.849	***0*.*009***
Physical	33.9 ± 1.48	8.8 ± 2.36	***0*.*000***	33.7 ± 1.87	7.9 ± 1.79	***0*.*000***	0.838	***0*.*009***
Cognitive	65.4 ± 3.22	14.7 ± 4.16	***0*.*000***	66.4 ± 2.90	13.3 ± 3.67	***0*.*000***	0.861	***0*.*008***
Psychosocial	36.0 ± 1.18	8.3 ± 2.67	***0*.*000***	36.8 ± 0.65	7.4 ± 2.49	***0*.*000***	0.878	***0*.*008***
**HADS**								
**Global score (0–42)**	18.0 ± 3.44	8.4 ± 0.93	***0*.*048***	19.9 ± 3.66	8.3 ± 1.99	0.055	0.942	0.173
Anxiety	9.4 ± 1.79	6.1 ± 0.48	0.238	10.0 ± 1.99	5.6 ± 1.03	0.166	0.982	0.368
Depression	8.6 ± 1.89	2.3 ± 0.75	***0*.*011***	9.9 ± 1.80	2.6 ± 1.16	***0*.*007***	0.781	0.062
**COMPASS-31**								
**Global score (0–100)**	34.0 ± 4.46	13.8 ± 2.06	***0*.*010***	62.7 ± 2.96	39.3 ± 1.77	***0*.*000***	0.918	***0*.*019***
Orthostatic intolerance	6.5 ± 0.96	1.6 ± 0.50	***0*.*002***	7.4 ± 0.62	1.1 ± 0.48	***0*.*000***	0.803	***0*.*018***
Vasomotor	1.8 ± 0.63	0.0 ± 0.00	***0*.*013***	2.2 ± 0.55	0.1 ± 0.13	***0*.*011***	0.649	0.116
Secretomotor	4.1 ± 0.60	3.7 ± 0.33	0.243	4.8 ± 0.51	0.6 ± 0.32	***0*.*001***	0.623	0.102
Gastrointestinal	10.1 ± 1.63	4.8 ± 1.13	***0*.*019***	13.4 ± 1.97	3.8 ± 1.00	***0*.*003***	0.775	0.053
Bladder	2.2 ± 0.63	0.6 ± 0.27	***0*.*040***	2.8 ± 0.66	0.1 ± 0.13	***0*.*001***	0.994	0.070
Pupillomotor	9.3 ± 1.37	3.1 ± 0.80	***0*.*003***	11.8 ± 1.00	1.9 ± 0.85	***0*.*001***	0.809	***0*.*020***
**SF-36**								
**Global score (0–100)**	26.6 ± 3.30	82.7 ± 3.52	***0*.*000***	95.4 ± 3.24	106.5 ± 0.93	***0*.*004***	0.942	***0*.*009***
Physical functioning	16.0 ± 5.52	85.0 ± 9.60	***0*.*002***	15.0 ± 3.50	98.1 ± 0.91	***0*.*000***	0.786	***0*.*018***
Physical role function.	0.0 ± 0.00	95.0 ± 3.33	***0*.*000***	0.0 ± 0.00	93.8 ± 4.09	***0*.*000***	0.857	***0*.*007***
Bodily pain	20.2 ± 5.60	84.8 ± 4.53	***0*.*000***	14.0 ± 4.64	91.3 ± 4.44	***0*.*000***	0.895	***0*.*008***
General health perception.	22.0 ± 4.55	74.1 ± 7.67	***0*.*001***	18.0 ± 3.27	81.5 ± 5.29	***0*.*000***	0.878	***0*.*012***
Vitality	14.0 ± 6.00	63.5 ± 4.48	***0*.*000***	7.0 ± 4.10	75.6 ± 6.30	***0*.*000***	0.988	***0*.*010***
Social role functioning	26.3 ± 8.00	93.8 ± 3.84	***0*.*000***	27.5 ± 7.17	95.3 ± 3.29	***0*.*000***	0.965	***0*.*009***
Emotional role functioning	59.9 ± 3.90	93.4 ± 4.40	0.075	63.3 ± 15.28	100.0 ± 0.00	0.050	0.794	0.283
Mental_health	54.4 ± 6.73	72.0 ± 3.86	***0*.*022***	50.8 ± 8.11	74.0 ± 4.84	0.055	0.924	0.158
**PSQI**								
**Global score (0–21)**	13.4 ± 0.85	4.4 ± 0.69	***0*.*000***	99.5 ± 17.81	30.7 ± 3.34	***0*.*002***	0.895	***0*.*016***
Subjective sleep quality	1.8 ± 0.39	0.4 ± 0.16	***0*.*011***	1.9 ± 0.31	0.6 ± 0.18	***0*.*008***	0.690	0.073
Sleep latency	2.1 ± 0.31	0.5 ± 0.17	***0*.*002***	2.1 ± 0.31	0.7 ± 0.29	***0*.*012***	0.758	***0*.*045***
Sleep duration	2.1 ± 0.31	1.5 ± 0.17	0.069	1.3 ± 0.37	1.1 ± 0.14	0.915	0.336	0.550
Habitual sleep efficiency	2.0 ± 0.42	0.3 ± 0.21	***0*.*004***	1.4 ± 0.43	0.1 ± 0.14	***0*.*042***	0.765	0.104
Sleep disturbances	2.1 ± 0.18	1.1 ± 0.10	***0*.*001***	2.1 ± 0.23	1.0 ± 0.00	***0*.*002***	0.964	***0*.*029***
Sleeping medication	1.5 ± 0.50	0.0 ± 0.00	***0*.*012***	1.5 ± 0.50	0.1 ± 0.13	0.060	0.848	0.192
Daytime dysfunction	1.8 ± 0.36	0.6 ± 0.22	***0*.*018***	1.7 ± 0.37	0.4 ± 0.18	***0*.*016***	0.894	0.101

Baseline self-reported outcome scores (global and subscales) of core symptoms, as explained in Patients and Methods section. Values are expressed as mean ± SEM for each item.

Season and group comparisons were carried out by Shreirer-Ray-Hare test (for non-parametric and two-way repeated measures ANOVA); season x group contrasts: U Mann-Whitney;

^a^Students’t test. Significant p-values (p) are represented in bold and italic.

Abbreviations: FIS-40, 40-item Fatigue Index Scale; HADS, Hospital Anxiety and Depression Scale; PSQI, Pittsburgh Sleep Quality Index; COMPASS-31, 31-items Abbreviated Composite Autonomic Symptom Score; SF-36, Medical Outcome Study 36-item Short Form Health Survey.

Participants were subjected to stringent exclusion criteria so as to remove any confounding comorbid conditions that might have influenced the data or the subject’s illness state. These exclusion criteria were: previous or current diagnosis of an autoimmune disorder, multiple sclerosis, psychosis, major depression, heart disease, hematological disorders, infectious diseases, sleep apnea or thyroid-related disorders; pregnancy or breast-feeding; smoking habit; strong hormone-related medications; and symptoms of CFS/ME that did not conform to the CFS/ME case criteria used for this study.

The mean duration of illness in the CFS/ME group was 8.4 ± 2.6 years. Patients reported no other fatiguing illnesses or primary psychiatric conditions that might explain the onset of their symptoms. In order to maintain functional status, patients’ medication was not withdrawn during the study, as this would undoubtedly have altered the expression of circadian rhythms. Nine patients were receiving analgesics (tramadol, paracetamol, tapentadol for generalized pain), five NSAIDs (ibuprofen, for generalized pain), six antidepressants (duloxetine, for neuropathic pain), four anticonvulsants (gabapentine, pregabaline, for neuropathic pain), and six anxiolytics (alprazolam, quetiapine and specially clorazepate dipotassicum, for general anxiety). No participants were taking either melatonin or hypnotics during the study.

Healthy controls were also recruited from the local population through advertisements inviting people without CFS/ME criteria to participate. Subjects were excluded if they had potential secondary causes for fatigue and/or autonomic dysfunction, or were taking medications that might cause fatigue or symptoms suggestive of autonomic dysfunction. All controls, but none of the CFS/ME patients, were in employment at the time of the study.

### Ethics statement

All participants gave written/signed informed consent to the research protocol after its approval by the Institutional Ethical Committee on Human Research of the Vall d’Hebron University Hospital under protocol reference number CEIC/23/01/HREC.

### General procedure

All eligible individuals went to the local hospital on Tuesday afternoons for a clinical assessment. Participants were asked to wear an ambulatory device, an actigraph (Act Trust^®^ from Condor Instruments, Sao Paulo, Brazil) on the wrist of the non-dominant arm continuously for seven days, except when showering or at the swimming pool. The same device was programmed to collect data on motor activity (accelerometer), skin temperature (°C) and light intensity (lux) at one minute intervals. These variables were recorded and stored in the device’s memory, for data analysis.

They were also asked to record the temperature of their environment, during the day and during the night, by means of a temperature sensor (IButton^®^ Thermochrom DS1921H) every 10 minutes. Subjects returned to the hospital after one week to return the devices and to undergo a second clinical assessment. Because each week only four subjects could be evaluated, the whole experiment lasted five weeks.

Subjects were also asked to complete a sleep diary where they recorded information on the time they went to bed, sleep onset and offset times, possible naps and any wakefulness during the scheduled sleep episode. The perception of sleepiness and mood were also evaluated on self-reported scales at three different times of day (9:00 AM, 3:00 PM and 9:00 PM) based on the Karolinska Sleepiness Scale (KSS) [31]. All these procedures were carried out at two different seasons of the same year: winter (data records from January-March) and summer (data records from June-September).

### Measures

Participants were also asked to provide complete validated self-report questionnaires of their current health status at one week of the first clinical assessment. Outcome measures, including changes in fatigue perception, sleep disturbances, anxiety and depression, autonomic symptoms and health-related quality of life, were assessed through self-report questionnaires completed by participants under the supervision of two trained investigators (JC-M and JA) who supervised compliance.

#### Fatigue perception

Fatigue was scored using the Fatigue Impact Scale (FIS-40), a 40-item questionnaire that includes three subscales reflecting the perceived effect of fatigue: cognitive (10 items), physical (10 items) and psychosocial functions (20 items) with each item being scored from 0 (no fatigue) to 4 (severe fatigue). The total score is calculated by adding together the responses to the 40 questions (range 0–160). Higher scores indicate more functional limitations due to fatigue [32].

#### Sleep quality

Sleep disturbances were assessed through the self-administered 19-item Pittsburg Sleep Quality Index (PSQI) questionnaire. Scores are obtained on each of seven components of sleep quality: subjective sleep quality, sleep latency, sleep duration, habitual sleep efficiency, sleep perturbations, use of sleeping medication, and daytime dysfunction. Each component is scored from 0 to 3 (0 = no sleep problems and 3 = severe sleep problems). The global PSQI score ranges from 0 to 21 points, with scores of ≥ 5 indicating poorer sleep quality [33].

#### Anxiety and depression

Anxiety and depression symptoms were scored using the Hospital Anxiety and Depression Scale (HADS), a validated self-reported tool composed of 14 items (seven related to anxiety symptoms and seven to depression). Each item on the HADS questionnaire is scored from 0–3 and so scores for each symptom range from 0 to 21. Scores of 0–7 are interpreted as normal, 8–10 as mild, 11–14 as moderate and 15–21 as severe for either anxiety or depression. The total HADS score ranges from 0 (no anxiety or depression) to 42 (severe anxiety and depression) [34].

#### Autonomic function

All participants were administered the Composite Autonomic Symptom Scale (COMPASS-31), a 31-item questionnaire designed to assess the frequency and severity of autonomic symptoms, grouped in six domains: orthostatic intolerance, vasomotor, secretomotor, gastrointestinal, bladder and pupillomotor systems. Added together, the six domain scores generate a total COMPASS-31 score of 0 to 100, with higher values indicating more severe autonomic symptoms [35].

#### Short Form 36-item Health Survey (SF-36)

The SF-36 questionnaire was used to assess health-related quality of life. This is a 36-item broadly-based self-report survey of physical and mental functioning status related to health. The SF-36 assesses functioning on eight subscales including domains of physical functioning, physical role functioning, bodily pain, general health perception, vitality, social role functioning, emotional role functioning, and mental health. Lower scores indicate a more negative impact of an individual’s health on functioning [36].

#### Sleep assessment

Sleep was measured via actigraphy and sleep diaries. The actigraph software, ActStudio, provided an estimation of the mean sleep latency, sleep onset, wake time, total sleep time and wake after sleep onset (WASO), sleep efficiency and number of awakenings for each subject. Sleep latency was defined as a length of time from bedtime to sleep onset time, and sleep efficiency as the percentage of total sleep time/total time in bed. The mean of these variables for the days of the study was used and then averaged per groups. Sleep variables were estimated by the algorithms of the software, which used the Cole-Kripke algorithm [37]. However, since CFS/ME patients reported being in bed without sleeping for longer than healthy controls, the onset of sleep was estimated by means of the time that lights were off according to the light intensity measured by the actigraph, and was recorded as an event marker in the actigraphic record. Thus for both groups the sleep analysis was carried out during the time that lights were off.

### Data analysis

Data from the actigraph—activity, skin temperature (DST, distal skin temperature) and light intensity—were analyzed with “El Temps, v293”, an integrated package for chronobiological analysis (A. Díez-Noguera, University of Barcelona, Spain). The rhythmic variables mesor, amplitude, acrophase were determined by adjusting the data to a 24h cosinusoidal curve. Thus, for each variable (activity, DST and light) we calculated: the mean 24-hour value (mean), the phase of the rhythm (acrophase) and the amplitude of the adjusted 24h rhythm (A_cos). Non-parametric circadian analysis was also performed as previously described [38,39]: M10 and L5 intervals denoted the 10 most active hours and the five least active respectively, and levels of activity during these two intervals were assessed. The non-parametric relative amplitude (RA_np) was calculated as follows: (M10 activity − L5 activity)/(M10 activity + L5 activity). In addition, the time of the mid L5 (L5c) and the distance between the middle of M10 and L5 (MLdis), the intradaily variability (IV), the stability of the rhythm (R), the percentage of variance explained by the 24h rhythm (PV) were also calculated. In the case of temperature, M5 and L10 were calculated instead of M10 and L5, due to the inverse pattern of this variable compared with activity.

In order to determine the times of the day during which the differences in activity and temperature were significant between groups, the 1440 min waveform of each individual was divided into 2h-stages and the mean per stage was calculated. Then, the mean value of each stage for each group of individuals and seasons was calculated. An ANOVA with repeated measures indicated that for each season, the group and the stages were significant factors (p<0.05 in all cases), as was the interaction between groups and stages. Then, post-hoc tests were carried out in the same model to examine differences between the groups for each stage.

Moreover, to study the pattern of the 24h waveform, a spectral analysis was carried out. Spectral analysis provides the main periodicities (harmonics) that shape the 24h rhythm. A Fourier analysis of the daily data was carried out for each individual, obtaining the phase and power content of the first 10 harmonics of the spectra. Then, values were averaged for the seven days of the recording, and the mean power spectrum of each individual was obtained. The values of the power of each harmonic (Hn, with “n” indicating the number of the harmonic) were used for comparisons between the groups: The higher the power of a harmonic, the higher its relevance in defining the pattern.

Statistically significant differences were tested using two-way repeated measures ANOVA. In general, “season” was the intra-subjects factor and “group” as the inter-subjects factor. Post-hoc tests were carried out by the Student’s t test to test differences between groups in each season and p-values below 0.05 were considered statistically significant after Bonferroni correction. In cases of non-parametric variables such as those obtained in the questionnaires, the repeated measures analysis was carried out by the Scheirer-Ray-Hare test [40] and to compare differences among the groups in each season, the Mann-Whitney *U* test was applied. Acrophases and other time values were compared by means of the Watson-Williams test for circular statistics. Moreover, to address the relationship between circadian variables and clinical features, the Pearson correlation was carried out. Data were analyzed using (IBM) SPSS Statistics version 22.

## Results

### Participants and clinical characteristics

As shown in Table 1, no differences were found in age or BMI between the groups. However, with regard to the clinical features assessed by self-reported outcome measures (**individual values in** S1 Table), statistically significant differences were found between groups on the global scores and on most of the subscales of the FIS-40, COMPASS-31, SF-36 and PSQI. In the case of HADS, differences were found between CFS/ME individuals and HCs for either overall scores or depression (p<0.05 for both). Moreover, for the SF-36 scale, only the emotional role functioning did not differ between groups.

### Environmental factors: Light and temperature

**Fig 1:**
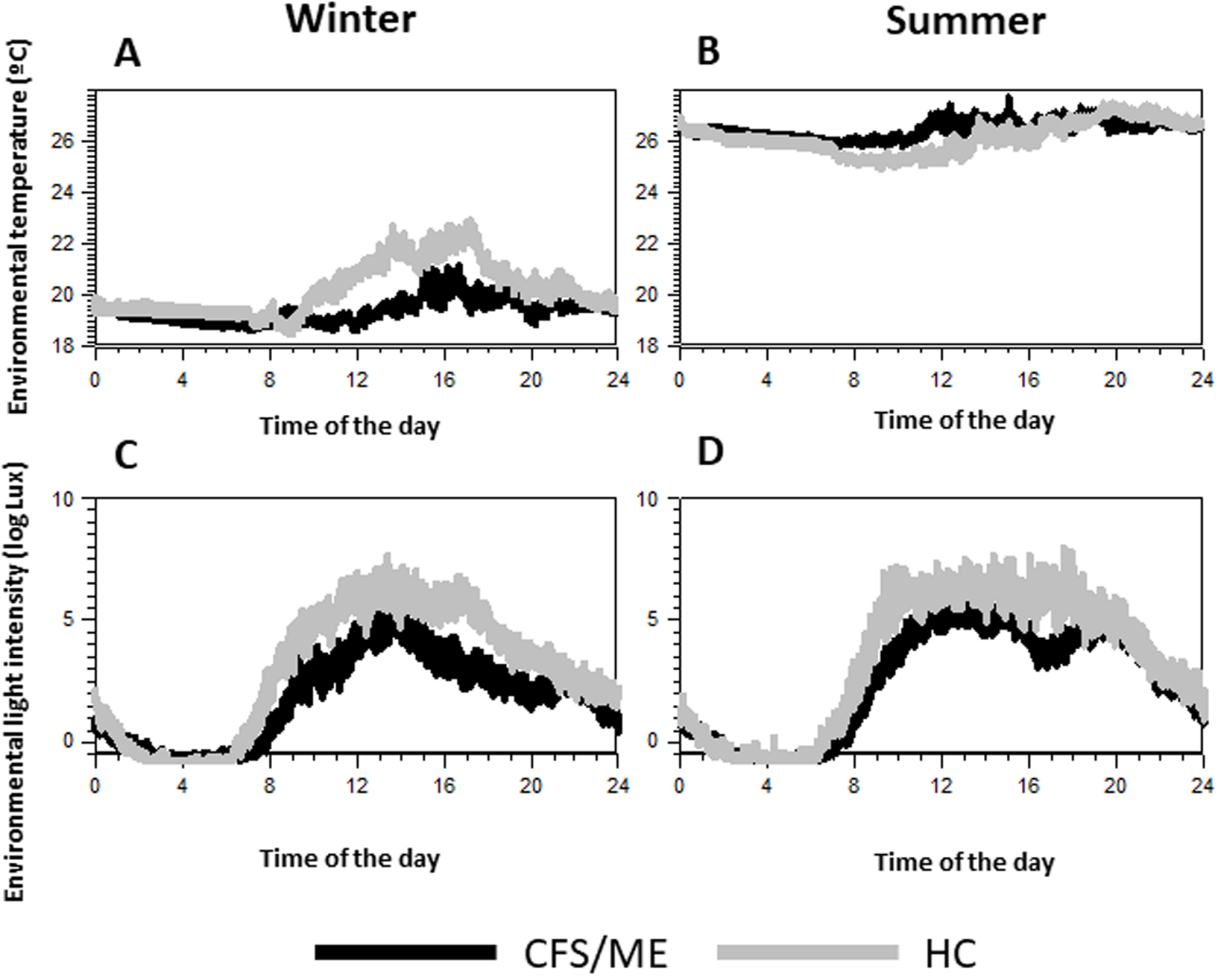
Mean ± SEM band of the environmental temperature and light intensity to which the subjects were exposed to in winter and summer. Black bands indicate the values registered for CFS/ME patients and grey bands the values for HCs.

Light and temperature change according to the season. Therefore, we analyzed these two factors to assess differences between the groups that might modify the data records. No differences were found in the mean environmental temperature between CFS/ME and HCs, nor in the lowest night-time values (Fig 1A and 1B). However, only in winter (Fig 1A), between 12:00 AM and 6:00 PM, healthy controls had higher environmental temperature than patients (22°C vs. 19°C). In summer (Fig 1B), temperature was naturally higher, but no differences between groups were observed during the day or at night.

With regard to light, light intensity and photoperiod length values were obviously higher. However, throughout the year, the mean and maximum value during the day were higher in the HCs than in the CFS/ME groups (Fig 1C and 1D).

### Subjective sleepiness and mood

**Fig 2:**
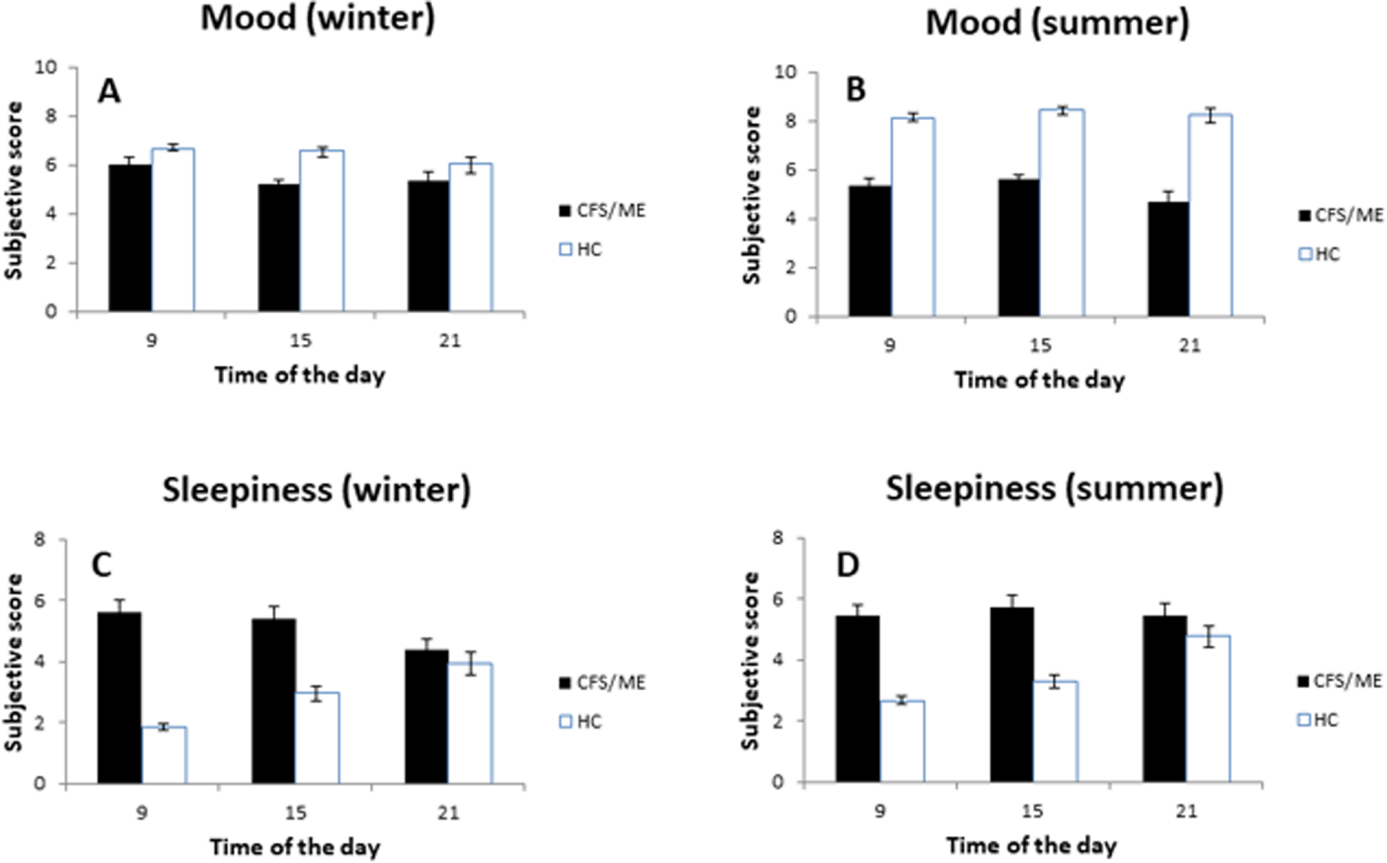
Mean values (± SEM) of self-reported tendency to sleep (A and B) and mood (C and D) for each group in winter and summer. Overall analysis indicated that sleepiness was higher in CFS/ME patients than in HCs (p = 0.001), while mood is lower in CFS/ME than in HCs (p = 0.009). HCs presented better mood in summer than in winter (p = 0.043). No overall effects of season or time of day were detected.

Subjective mood and the tendency to sleep assessed at three time points in the day, presented different values in the two groups (Fig 2). A two-way repeated measures ANOVA for season and time of day indicated that mood (Fig 2A and 2B) was always lower in CFS/ME than in HCs (p = 0.009). In contrast, ANOVA indicated more sleepiness in CFS/ME patients than in HCs (p = 0.001) (Fig 2C and 2D). No overall effects of season or time of day were detected, although post-hoc tests indicated that HCs presented better mood in summer than in winter (p = 0.043).

### Sleep diary

CFS/ME patients and HCs reported different sleeping patterns. HCs reported getting to sleep after a mean time of 8 min, while CFS/ME reported being in bed for between 1 to 3 hours before going to sleep (mean subjective latency ± SEM: 93 ± 25 min). Although time in bed differed between groups, subjective sleeping time did not (Table 2). In winter, HC did not report napping, however several CFS/ME patients did over the seven days (two patients reported one day of napping, one patient two days, one patient three days and one patient four days). In summer, one HC reported napping half an hour for four days. CFS/ME patients slept a mean of 1.5 h (two patients one day of napping, one patient three days and two patients almost daily). Participants did not report significant differences in their schedules between weekdays and the weekend.

**Table 2:** Sleep variables measured by the sleep diary and actigraphy in winter and summer.

Variable	Winter			Summer				
	CFS/ME	HC	p.	CFS/ME	HC	p.	p	
	n = 10	n = 10		n = 10	n = 8		season	group
**Sleep Diary**								
Time to bed (h:min ± min)^a^	23:30 ± 1	00:09 ± 13	0.525	23:58 ± 31	00:26 ± 18	0.487	0.528	0.362
Latency (min)	93.7 ± 25.6	8.50 ± 1.1	***0*.*022***	74.0 ± 20	7.1 ± 1.1	***0*.*011***	0.287	***0*.*013***
Wake time (h:min ± min) ^a^	08:28 ± 20	07:19 ± 8	***0*.*010***	08:58 ± 22	07:59 ± 14	***0*.*018***	0.223	***0*.*001***
Time in bed (min)	543 ± 33	429± 11	***0*.*007***	531 ± 12	428± 11	***0*.*007***	0.753	***0*.*003***
Sleep Time (min)	457 ± 46	420 ± 11	0.464	456 ± 28	420 ± 10	0.304	0.931	0.372
**Actigraphy**								
Sleep efficiency (%)	86.7 ± 2.23	91.3 ± 1.86	0.089	88.1 ± 1.23	89.1 ± 1.86	0.659	0.653	0.114
Time in bed (min)	566 ± 38	463 ± 13	***0*.*014***	539 ± 27	449 ± 11	***0*.*012***	0.397	***0*.*006***
Number of awakenings	10.4 ± 1.62	7.1 ± 1.69	0.060	9.9 ± 1.15	8.0 ± 1.42	0.320	0.488	0.065
Latency (min)	8.2 ± 0.9	5.6 ± 1.4	0.207.	9.6 ± 2.0	6.9 ± 1.6	0.319	0.235	0.217
WASO (min)	72.0 ± 18.0	31.0 ± 7.1	***0*.*039***	52.6 ± 5.6	39.6 ± 7.4	0.171	0.807	***0*.*011***
WASO/hour (min)	7.3 ± 1.5	4.0 ± 0.9	0.055	5.9 ± 0.6	5.3 ± 1.0	0.624	0.825	0.059

Values are expressed as mean ± SEM.

Season and group comparisons were carried out by two factor repeated measures ANOVA; season x group contrasts: Students’t test,

^a^ Watson-Williams test. Significant p-values (p) are represented in bold and italic.

### Sleep parameters measured by the actigraph

To study sleep efficiency with the actigraph, we defined time in bed on the basis of event markers: the first event, sleep onset, coincided with lights off according to the light measured by the actigraph, and the second event, sleep offset, when lights were on. According to these events, the results (Table 2) indicate that CFS/ME patients had a longer time in bed but that their sleep efficiency was shorter and they presented more awakenings. WASO differed between groups, but when it was defined per hour of sleep, no differences were found (p = 0.06).

### Circadian analysis of actigraphic measures (activity and DST)

**Fig 3:**
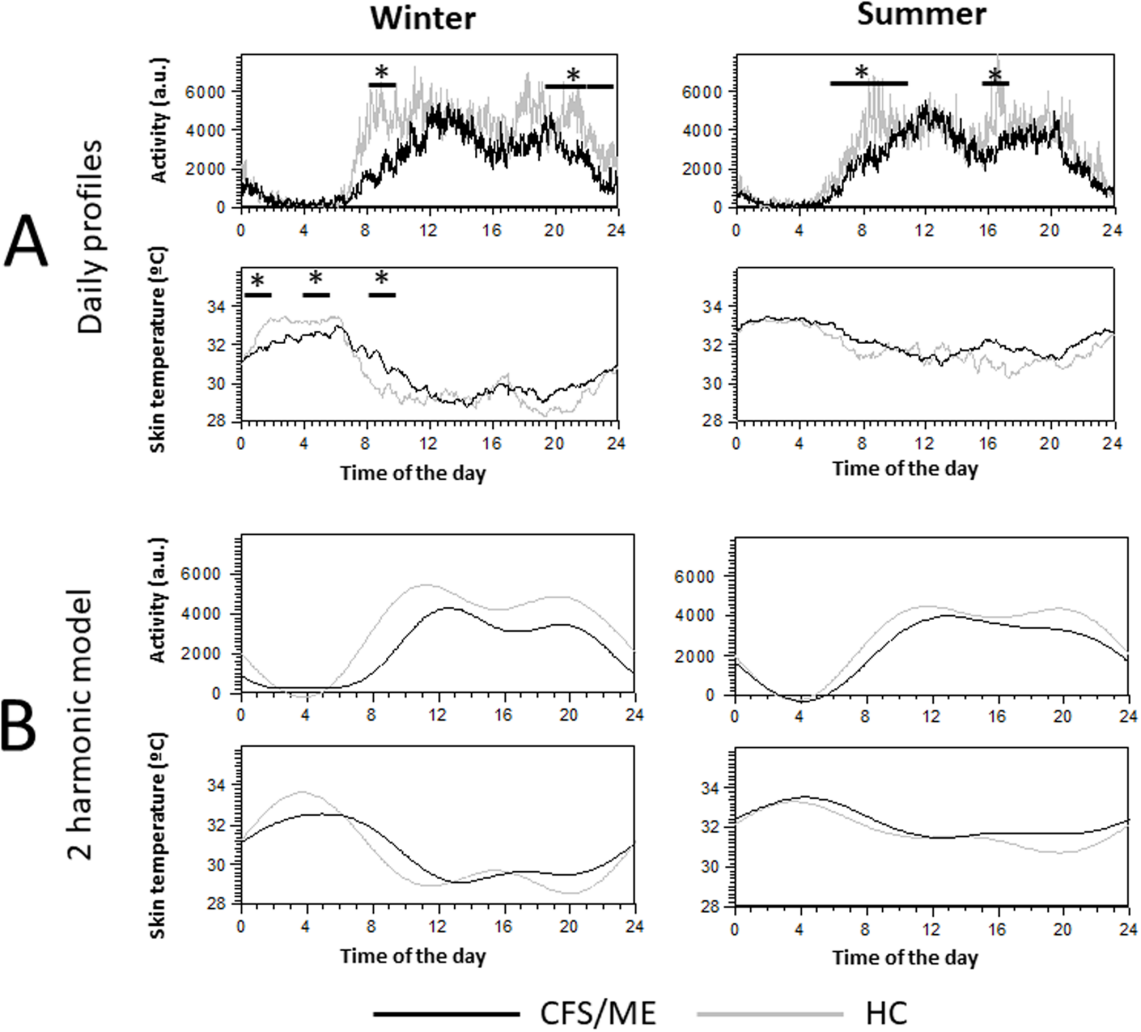
24-hour profiles (mean for each group) of activity and temperature for HC and CFS/ME subjects in winter and summer. **A**. Mean daily profiles of activity and skin temperature. Dark lines correspond to CFS/ME patients and grey lines to HCs (to aid visualization, SEM is not shown). Asterisks indicate differences between groups at p<0.05 (see text, segments analysis). **B**. Synthesis of the mean waveform for activity and skin temperature, according to the first two harmonics of the spectrum (see text, spectral analysis subsection).

The double plotted actograms of the individuals did not reveal differences between workdays and weekend (supplementary information, S1 Fig). The daily profiles of activity and skin temperature indicate differences between the groups and the seasons (Fig 3A).

The analysis of the circadian variables (Table 3; individual values in S1 Table) indicates that in the case of activity, the mean values were significantly higher in HCs than in CFS/ME, as were the maximum values during the day (M10). As regards skin temperature, the maximum nocturnal values (M5) were lower in CFS/ME than in HCs in winter (p = 0.02).

**Table 3:** Rhythmic variables for activity and skin temperature in winter and summer.

Variable	Winter			Summer				
	CFS/ME	HC	p	CFS/ME	HC	p	p	
	n = 10	n = 10		n = 10	n = 8		season	group
**Activity**								
mean (a.u.)	2192 ± 198	3146 ± 193	***0*.*003***	2289 ± 180	2791 ± 185	0.072	***0*.*045***	***0*.*008***
Acrophase (min) ^a^	931 ± 33.9	912 ± 21.8	0.633	937 ± 28.9	932 ± 21.5	0.899	0.610	0.624
A_cos (a.u)	2125 ± 203	2397 ± 182	0.333	2059 ± 160	2163 ± 136	0.639	***0*.*013***	0.287
R_cos (a.u.)	1.0 ± 0.03	0.8 ± 0.03	***0*.*000***	0.9 ± 0.04	0.8 ± 0.04	***0*.*046***	0.210	***0*.*002***
RA_np (a.u.)	0.9 ± 0.01	0.9 ± 0.01	0.427	0.9 ± 0.01	0.9 ± 0.02	0.744	0.324	0.810
IV	0.4 ± 0.02	0.4 ± 0.02	0.077	0.4 ± 0.02	0.4 ± 0.02	0.172	0.327	0.162
R	1.0 ± 0.01	1.0 ± 0.01	0.349	1.0 ± 0.01	1.0 ± 0.01	0.813	0.618	0.522
PV (%)	34.9 ± 1.34	38.8 ± 1.98	0.120	33.9 ± 1.32	39.5 ± 3.96	0.163	0.842	***0*.*045***
M10 (a.u.)	3859 ± 337	4855 ± 304	***0*.*042***	3895 ± 290	4371 ± 247	0.244	***0*.*019***	0.058
L5 (a.u.)	123 ± 15.7	134 ± 14.8	0.599	120 ± 12.4	160 ± 41.9	0.322	0.458	0.392
L5c (min) ^a^	273 ± 29	245 ± 14	0.372	249 ± 21	248 ± 15	0.973	0.627	0.474
MLdis (min) ^a^	654 ± 23	701 ± 36	0.262	773 ± 29	769 ± 27	0.929	***0*.*002***	0.547
Espectral analysis								
Power H1 (%)	19.0 ± 1.28	16.8 ± 1.60	0.311	17.3 ± 1.08	17.1 ± 1.47	0.923	0.068	0.849
Phase H1 (min) ^a^	931 ± 34	912 ± 22	0.621	937 ± 29	930 ± 22	0.849	0.622	0.586
Power H2 (%)	2.6 ± 0.78	6.1 ± 0.77	***0*.*005***	3.3 ± 0.81	5.0 ± 0.91	0.195	0.759	***0*.*027***
Phase H2 (min) ^a^	274 ± 89.8	567 ± 19.8	***0*.*022***	533 ± 77.2	583 ± 19.3	0.597	0.330	0.063
Power H3 (%)	1.7 ± 0.46	0.8 ± 0.28	0.132	1.7 ± 0.40	1.2 ± 0.55	0.459	0.241	0.290
Phase H3 (min) ^a^	39 ± 67	142 ± 35	0.192	204 ± 46	126 ± 45	0.266	0.174	0.941
**Temperature**								
mean (°C)	30.5 ± 0.30	30.5 ± 0.16	0.993	32.2 ± 0.22	31.5 ± 0.51	0.180	***0*.*000***	0.238
Acrophase (min) ^a^	321 ± 31.0	226 ± 26.1	0.775	242 ± 40.1	138 ± 71.1	0.266	0.562	0.498
A_cos (a.u)	1.9 ± 0.39	2.0 ± 0.14	0.712	1.0 ± 0.18	1.4 ± 0.49	0.402	***0*.*015***	0.358
R_cos (°C)	0.1 ± 0.01	0.1 ± 0.00	0.770	0.0 ± 0.01	0.0 ± 0.02	0.376	***0*.*019***	0.362
RA_np (°C)	0.1 ± 0.01	0.1 ± 0.00	0.351	0.0 ± 0.00	0.0 ± 0.01	0.270	***0*.*010***	0.144
IV	0.0 ± 0.00	0.0 ± 0.00	0.636	0.0 ± 0.00	0.0 ± 0.00	0.230	0.412	0.966
R	0.9 ± 0.04	0.9 ± 0.02	0.091	0.8 ± 0.06	0.7 ± 0.09	0.226	***0*.*017***	0.656
PV (%)	41.0 ± 5.50	48.1 ± 3.50	0.285	38.9 ± 5.09	36.8 ± 9.36	0.835	0.291	0.601
M5 (°C)	32.6 ± 0.23	33.4 ± 0.23	***0*.*021***	33.4 ± 0.16	33.2 ± 0.24	0.537	0.060	0.217
L10 (°C)	29.0 ± 0.57	29.0 ± 0.25	0.990	31.5 ± 0.31	30.4 ± 0.90	0.237	***0*.*002***	0.319
L5c (min) ^a^	902 ± 46	1014 ± 45	0.097	968 ± 44	750 ± 72	***0*.*044***	0.271	0.832
MLdis (min) ^a^	575 ± 41	792 ± 47	***0*.*004***	689 ± 45	649 ± 57	0.611	0.880	0.061
Espectral analysis								
Power H1 (%)	25.8 ± 5.77	22.2 ± 2.20	0.568	21.6 ± 4.01	18.9 ± 8.21	0.756	0.354	0.725
Phase H1 (min)^a^	225 ± 35	236 ± 15.4	0.770	242 ± 40.1	140 ± 71.8	0.276	0.312	0.210
Power H2 (%)	5.1 ± 1.33	14.2 ± 3.22	***0*.*018***	6.0 ± 2.18	6.9 ± 1.95	0.765	0.135	***0*.*028***
Phase H2 (min) ^a^	302 ± 31	201 ± 30.5	***0*.*028***	290 ± 33.0	193 ± 30.2	0.052	***0*.*020***	***0*.*002***
Power H3 (%)	3.5 ± 0.88	2.2 ± 0.77	0.293	3.1 ± 1.03	1.6 ± 0.73	0.259	0.463	0.090
Phase H3 (min) ^a^	25 ± 32	48.1 ± 44.49	0.671	68 ± 28	57 ± 48	0.943	0.531	0.856

Values are expressed as mean ± SEM.

Season and group comparisons were carried out by two factor repeated measures ANOVA; season x group contrasts: Students’t test,

^a^Watson-Williams test. Significant p-values (p) are represented in bold and italic.

**Abbreviations**: A_cos: Amplitude of a 24h cosinusoidal curve; R_cos: Relative amplitude; RA-np: non-parametrical relative amplitude; V: intradaily variability; R: Rayleigh test; PV: Variance (%) explained by the 24h rhythm; M10, L5; L5c, MLdis (see text for explanation).

### Segments analysis

The mean value of each 2h stage was calculated for each group and season. A linear model with repeated measures indicated that group and stage were significant factors for each season (p<0.05 in all cases), but also highlighted the interaction between the groups and the stages. Therefore, post-hoc tests were carried out to examine differences between the groups for each stage. In winter HCs presented higher values than CFS/MEs for activity between 8:00 AM and 10:00 AM, and between 8:00 PM and 12:00 AM (p<0.05 in all cases). In summer, activity was higher in HC between 05:00 to 10:00 AM and between 4:00 to 6:00 PM. In the case of temperature, it was higher in HCs between 12:00 and 2:00 AM, from 4:0 to 6:00 AM and from 8:00 to 10:00 AM. In summer, skin temperature did not differ between groups in any of the segments (see Fig 3A).

### Spectral analysis

To compare the 24h pattern of the two groups, we assessed the power content of the different harmonics of the spectrum (Table 2, spectral analysis: **individual values in** S1 Table). The power and the phases of the first three harmonics of the spectrum were compared. The most important results were that power of H2 (the power of the 12h rhythm) was higher in HCs than in CFS/ME (p<0.05) for both temperature and activity profiles and that phases of this harmonic also differed between groups. Thus, the characteristic rhythmic pattern of both groups can be reconstructed with a two-harmonic model (Fig 3B) which allows easy comparison of the profiles.

### Correlations between rhythmic variables and outcome measures

To explore possible relationships between variables we carried out correlation analysis between the rhythmic variables and the outcome measures (clinical features), including the correlations for each domain of the questionnaires. The statistical significance for multiple tests was corrected considering Bonferroni’s correction and the probability that for the same rhythmic variable, n correlations were statistically significant (S2 Table). In this way, the individual α value for maintaining the overall probability α_n_ equal or below 0.05 is: α = 1-(1-α_n_^1/n^)^1/N^ = 1-(1–0.05^1/n^)^1/N^, where N is the total number of correlations for one variable and n the number of significant ones (p<0.05). For example, in the case of a variable whose correlation is tested with 31 other variables (N), the Bonferroni correction gives an individual α = 0.0016, but if correlations with six different variables (n) are significant (p<0.05), this correction sets α at 0.0297. This correction was made for each rhythmic variable in its comparison with the clinical features.

Considering all the values (those for summer and winter and for both groups), the rhythmic variables that correlated best with most of the clinical features were: for activity, the mean, R_cos, IV, PV, and P2; for temperature P2. In all cases, they correlated negatively with FIS-40, HADS, COMPASS-31, PSQI and positively with SF-36 (values in S2 Table). When the analysis was carried out separately for both seasons certain differences were observed.

In winter, for all the participants: a) with regard to activity, mean activity correlated negatively with FIS-4, PSQI, COMPASS-31 (global and all domains excepting vasomotor and secretomotor) and positively with SF-36. M10 correlated negatively with FIS-40, COMPASS-31, PSQI and positively with SF-36; b) with regard to temperature, M5 correlated negatively with PSQI, and positively with SF-36 (bodily pain, physical and social role functioning subscales). When the correlations of the power of the harmonics of the spectrum with the clinical features were tested, P1 did not correlate, but P2 correlated negatively with FIS-40, PSQI and COMPASS-31, and positively with most subscales of SF-36, for both activity and temperature.

In summer, mean activity, R_cos and IV were the variables that correlated with the clinical features. Mean activity correlated negatively with FIS_40 (global and all subscales) and positively with SF-36 (global, and vitality). R_cos correlated negatively with FIS_40 (global and all subscales) positively with SF-36 (global, physical functioning and bodily pain) and positively with COMPASS-31 (global). As regards the skin temperature in summer, none of the variables correlated with clinical features at the level of significance defined.

## Discussion

This study provides the first evidence that the circadian profile is altered in CFS/ME patients and that this rhythmic pattern, specifically measured by the manifestation of the second harmonic of the power spectrum, could be an indicator of this condition. One of the limitations of this exploratory study is the small sample size: however, the groups were well defined, based on the assessment of the clinical features by a CFS/ME specialist and the responses to the psychological and wellbeing questionnaires, and can therefore be considered representative. Moreover, this is the first report to study circadian variables and their correlation with clinical features and at two different times of the year. The fact that the study was held in summer and winter with the same subjects allowed us to record the variables that were relevant for the group, and to detect circadian variables that might be masked by the different environmental conditions in the two seasons.

All the participants in the present study maintained relatively regular sleep habits, which varied little between weekends and weekdays. However, the sleep schedules reported in the sleep diaries indicated that the CFS/ME group stayed in bed longer, probably as a result of their fatigability; this complicated the assessment of sleep time by actigraphy. Therefore, when using actigraphy, the lights on/off pattern for each individual proved very useful for comparing the sleep pattern. As for the light pattern, although CFS/ME group reported more sleep disturbances, other variables such as latency, fragmentation of the rest-activity cycle and stability did not differ significantly with respect to the control group. These findings highlight the importance of using light exposure recording in the sleep studies in these patients.

A decrease in activity level is a cardinal feature of many proposed definitions of CFS/ME, which produces a substantial reduction or impairment in the ability to carry out daily activities of life. In fact, a reduction in activity level of at least 50% below premorbid levels has been reported [2] Using actigraphy, activity levels during the day were found to be lower in patients than in controls, as has already been reported [24]. Our results confirm these differences and add that between 12h and 14h, activity did not differ between the groups. Since it has been reported that graded exercise markedly improves symptoms and activities of daily living in CFS/ME patients [41], we suggest that determining the time of day when actigraphic activity shows the highest values might help to indicate the best time for these patients to schedule their activities.

Distal skin temperature shows practically an inverted curve with respect to activity. The DST profile has been well described [42], with highest values at night and lowest during the day, but with a secondary peak in the afternoon (nap peak or post-lunch peak). Moreover, DST in the late evening, between 7:00 PM and 9:00 PM, exhibits minimum values that correspond to the “wake maintenance zone” at the beginning of the night period. In our CFS/ME sample this decrease is not observed. Modifications in the wake maintenance zone have been associated with aging [43], a finding that corroborates the impairment in the patients, and reinforces the importance of studying the entire daily profile of the variables.

We also observed that CFS/ME patients showed lower DST values at night than controls. This finding corroborates those of other studies which have shown that the core body temperature is higher at night in adolescents with CFS/ME [15], and supports the idea of a hypothetical autonomic dysregulation linking vascular dynamic alterations and CFS/ME. Actually, vasoconstrictor response to cold is impaired in CFS/ME patients [44] suggesting impairment in the sympathetic responses and thermoregulatory disturbances in this condition. However, other studies [16] have not shown differences in the core body temperature rhythm of CFS/ME patients, but rather a disruption of the expected correlation between the timing of melatonin onset and body temperature acrophase, suggesting a dissociation of the circadian regulation [19]. This difference in DST was not found in summer, since skin temperature was high in both groups. This may be due to the possible masking effect of the high environmental temperature at this time of the year. This finding highlights the need to record the environmental temperature DST simultaneously and the need to indicate the season when carrying out actigraphic recordings. Activity also changed between seasons, with HCs showing lower values in summer than in winter, but the profile was maintained for both groups.

In spite of the difference between the two groups in the time in bed and employment status (which probably reduces the degree of routines during the day and alters circadian and sleep patterns), no differences in the acrophase of the 24h rhythm were found for either activity or temperature. Studies of human circadian rhythms have shown that under the normal 24h rhythm, the circadian pattern is not purely sinusoidal, but are also modeled by a 12h component, and that post-lunch variations are linked to human physiology [45]. The post-lunch component presents as a dip in activity and a peak in DST, which is manifested more clearly if individuals nap, and is reflected in the power of the second harmonic of the spectrum. Although Fourier analysis should be interpreted with care, it is a useful tool for indicating the complex regulation of the circadian system. It is worth noting that CFS/ME patients showed a different spectrum to the control group, which was mainly characterized by a less manifested second harmonic. Unlike controls, CFS/ME patients did not display a clear increase in the post-lunch temperature. This finding is interesting, because some of the patients reported napping some days while HCs did not.

Sleep wake cycle and thermoregulation are strongly interrelated since people tend to go to bed when the core body temperature decreases, due to heat loss and the increase in the distal skin temperature. Since sleep in any circadian phase induces vasodilatation mainly in distal skin regions [46,47], the lack of a post-lunch increase in DST may indicate a dysregulation of this mechanism in these patients. It should also be noted that it is precisely in the post-lunch period when temperature values in CFS/ME patients correlate positively with activity, something that does not occur in HCs.

Since circadian rhythm variations in peripheral tissues are mainly due to the balance between sympathetic and parasympathetic systems, we may consider whether there might be an imbalance between these systems in CFS/ME, perhaps due to a sympathetic hyperactivation [44]. The skin sympathetic system activity seems to play a crucial role in blood flow regulation in the skin [48]; thus, the lower values of DST at night, and in the post-lunch hours in the CFS/ME patients may indicate an abnormal thermoregulation which might be useful biomarker for diagnosis.

Our results also showed that CFS/ME patients were exposed to lower light intensity during the day than HCs. CFS/ME patients have reported abnormal light sensitivity as a symptom of abnormal visual intolerance to light, or photophobia [49]. However, we need to explore whether exposure to low light intensity is voluntary or is due to the fact that individuals spend more time indoors. Light is an important factor for the synchronization of the circadian rhythms and can also impact mood and cognition [50]. Thus, low intensity levels during the day may alter sleep during the following night [51] and may contribute to the exacerbation of symptoms of the CFS/ME. Although, bright light phototherapy in these patients has been shown not to be beneficial [52], some studies in women with fibromyalgia [53] have indicated that bright light during the morning improves pain sensitivity, suggesting that light therapy might be an acceptable adjuvant approach that merits further investigated.

Although our study clearly indicates different circadian patterns in CFS/ME patients that may suggest autonomic dysfunction, certain limitations of the study need to be considered. The first is its necessarily empirical approach; to our knowledge, very few reports have studied circadian rhythms with actigraphy to simultaneously measure activity, skin temperature and light exposure in CFS/ME patients, and none have analyzed the ultradian structure of the daily profile of these variables. This obliged us to explore the different variables that might indicate the diagnosis and perhaps guide prognosis later on. Moreover, because of the low number of participants we cannot extrapolate the results to the whole CFS/ME population; studies in severely ill CFS/ME individuals are now urgently required.

Furthermore, although actigraphy is considered by the American Association of Sleep Medicine as an objective method for assessing circadian rhythm-associated sleep/wake disorders [54], and is strongly correlated with polysomnography, it is not always accurate for distinguishing periods of still wakefulness from periods of sleep. The light pattern should also be contrasted with the actigraphic records, as we did in this study.

Circadian rhythms govern many physiological functions including the sleep/wake cycle, alertness/tiredness, body temperature, thermoregulatory changes in skin blood flow, or intellectual performance and their misalignment may be a risk for health [55,56]. Our study highlights the importance of using an objective measure such as actigraphy in addition to self-assessments in order to establish a “circadian signature” in subgroups of CFS/ME subjects.

The current study is the first to correlate patient-reported outcome measures with rhythmic variables. Importantly, some of the circadian variables correlated with clinical outcome measures, indicating the objective value of actigraphy for diagnosis. The variables that negatively correlated with FIS-40 and HADS and positively with SF-36 can be considered good indicators of the patient’s health status: for instance, the mean, the relative amplitude and the intra-daily variability in the case of activity, or the nocturnal value of skin temperature in winter. Interestingly, the power of the 12h rhythm, in both variables, highly correlated with the clinical features. However, the consequences of the variable used to measure circadian rhythms cannot be ignored. As observed, the circadian rhythm of activity reported group differences regardless of the season, but in the case of skin temperature, since it is a variable sensitive to the environmental temperature, it was not adequate in the case of high environmental temperature, such as those that occured in summer.

It remains to be clarified whether or not alterations of the circadian clock are a feature of CFS/ME, but it is worth stressing that a previous study found an association between the NPAS2 gene and circadian regulation alterations in CFS/ME [57]. The identification of molecular clock (circadian) gene polymorphisms and the features of circadian rhythm alterations might be useful in CFS/ME, therefore, the circadian system could be a possible therapeutic target to improve the quality of life of these patients. The restoration of the rest–activity rhythm and temperature are probably not just passive reflections of the clinical evolution, but also play an active role in recovery in these patients.

Investigation of the relationship between the clinical symptoms and chronobiological effects in CFS/ME is likely to aid diagnosis and broaden our understanding of the pathophysiology of the condition.

## Conclusions

This study provides important information on the mechanisms implicated in dysautonomia from a chronobiological perspective. The relationship between circadian rhythms and energy metabolism (circadian metabolomics) is just beginning to shed light on some of the mechanisms underlying circadian behavior and physiology, and much remains to be gained by experiments of the kind described here. Further studies using actigraphy as a non-invasive tool, including skin temperature and bright-light exposure analysis, are needed to evaluate circadian disruptions in different subtypes of CFS/ME subjects, including severely ill ME/CFS individuals (housebound and bedbound patients). These findings suggest novel potential strategies of management and behavioral interventions to prevent rhythm disturbances in this population. The opportunity to discover non-invasive, objective biomarkers, to better predict physiological time, and to develop novel insights for personalized medicine are key areas in which circadian metabolomics experiments are likely to be extremely valuable in the near future in CFS/ME. These experiments are likely to have an important bearing on the therapeutic applications of circadian rhythms for this condition.

## Supporting information

S1 Table Individual values of the rhythmic variables and clinical features of the study participants. (XLSX)

S2 Table Pearson correlation coefficient and their statistical significance between rhythmic variables and patient-reported outcome measures. (PDF)

S1 Fig Double-plotted actograms in summer and winter of each study participant. (TIF)
