# Supplementary material for: Circadian rhythm abnormalities and autonomic dysfunction in patients with Chronic Fatigue Syndrome/Myalgic Encephalomyelitis
Source: PLoS One. 2018 Jun 6;13(6):e0198106. doi: 10.1371/journal.pone.0198106 (PMC5991397; doi:10.1371/journal.pone.0198106)
Supplement: S2 Table — (PDF) [file pone.0198106.s002.pdf]

SUPPLEMENTARY DATA: CORRELATION BETWEEN RHYTHMIC VARIABLES AND CLINICAL FEATURES

Significant correlations have been calculated considering Bonferroni's correction and the probability to obtain n signficant correlations at p<0.05 for the same variable.

According with the Bonferroni criteria, in a set of N tests, the probability of obtaining a significant result just by chance is $\alpha_1 = 1-(1-\alpha)^{1/N}$. In order to keep the global risk at $\alpha_1 = 0.05$, one must reduce the individual risk at $\alpha = 1-(1-\alpha_1)^{1/N} = 1-(1-0.05)^{1/N}$ which is very well approached (for $\alpha_1<0.1$) by $\alpha = \alpha_1/N = 0.05/N$. On the other hand, one must consider that fact taht a single variable can cumulate n significant correlations with other variables. Following the same rational, the probability of getting this result by chance in a set of N tests is $\alpha_n = (1-(1-\alpha)^N)^n$. In this case the individual α value to keep the global probability α_n equal or below 0.05 will be $\alpha = 1-(1-\alpha_n^{1/n})^{1/N} = 1-(1-0.05^{1/n})^{1/N}$, being this the p-value we have used to test the correlation coefficients. For example, in the case of a variable which correlation is tested with 31 other variables the Bonferroni correction gives an individual α =0.0017, but considering the possible correlation with 6 different variables, this correction sets α in 0.0297.

Season: Winter. Variable: activity. Subjects: All

VARIABLE (Activity or Distal Skin Temperatureerature)	SUBJECTS	RHYTHMIC VARIABLE		Scorefis40_1	Scorefis40_2	Scorefis40_3	All _fis40	ScoreHADS_1	ScoreHADS_2	All _HADS	C31_OI	C31_vasom	C31_secremot	C31_GI	C31_Bladder	C31_pupilmot	C31_All
Activity	All (n=20)	mean 24h	Pearson correlation coeficient	-,723**	-,701**	-,720**	-,718**	-.193	-.424	-.344	-,574**	-.163	-.317	-,508*	-,609**	-,560*	-,603**
			Sig. (bilateral)	.000	.001	.000	.000	.416	.062	.137	.008	.493	.173	.022	.004	.010	.005
Activity	All (n=20)	A_cos	Pearson correlation coeficient	-.397	-.341	-.375	-.367	.058	-.128	-.048	-.230	.193	-.256	-.204	-,476*	-.219	-.251
			Sig. (bilateral)	.083	.142	.103	.112	.806	.591	.839	.329	.415	.277	.387	.034	.354	.286
Activity	All (n=20)	R_cos	Pearson correlation coeficient	,696**	,742**	,692**	,724**	,472*	,585**	,573**	,710**	,600**	.179	,623**	.359	,667**	,714**
			Sig. (bilateral)	.001	.000	.001	.000	.036	.007	.008	.000	.005	.451	.003	.120	.001	.000
Activity	All (n=20)	RA_np	Pearson correlation coeficient	-.214	-.113	-.186	-.159	.137	.066	.105	-.295	-.199	-.145	-.299	-,584**	-.043	-.288
			Sig. (bilateral)	.364	.635	.433	.504	.565	.782	.661	.207	.400	.542	.200	.007	.857	.219
Activity	All (n=20)	IV	Pearson correlation coeficient	-.362	-.407	-.366	-.389	-.338	-.359	-.375	-.144	-.164	.084	.091	.159	-.263	-.077
			Sig. (bilateral)	.116	.075	.113	.090	.145	.120	.103	.544	.489	.725	.703	.504	.262	.748
Activity	All (n=20)	R	Pearson correlation coeficient	-.350	-.369	-.327	-.356	.132	-.173	-.040	-.158	-.071	-.099	-.103	-.403	-.076	-.160
			Sig. (bilateral)	.130	.110	.159	.123	.580	.466	.866	.507	.765	.677	.665	.078	.749	.501
Activity	All (n=20)	PV	Pearson correlation coeficient	-.335	-.314	-.290	-.316	.072	-.047	.006	-.219	-.284	.172	-.240	-.304	-.043	-.195
			Sig. (bilateral)	.149	.178	.214	.175	.764	.842	.981	.354	.225	.469	.309	.193	.856	.410
Activity	All (n=20)	M10	Pearson correlation coeficient	-,598**	-,551*	-,577**	-,575**	-.066	-.289	-.204	-.430	.013	-.299	-.373	-,565**	-.402	-,447*
			Sig. (bilateral)	.005	.012	.008	.008	.781	.217	.389	.059	.956	.200	.105	.009	.079	.048
Activity	All (n=20)	L5	Pearson correlation coeficient	-.148	-.244	-.190	-.208	-.192	-.272	-.254	.010	.160	-.036	.019	.232	-.255	-.031
			Sig. (bilateral)	.535	.299	.422	.379	.417	.246	.280	.966	.501	.881	.937	.324	.278	.897
Activity	All (n=20)	P1_A	Pearson correlation coeficient	.098	.119	.011	.086	.216	.015	.112	-.009	.259	-.227	.152	-0.1134	.029	.052
			Sig. (bilateral)	.680	.617	.963	.719	.360	.950	.638	.971	.270	.336	.523	.634	.905	.828
Activity	All (n=20)	P2_A	Pearson correlation coeficient	-,460*	-,469*	-.440	-,463*	-.129	-.309	-.245	-,536*	-,659**	-.027	-,650**	-.290	-.403	-,582**
			Sig. (bilateral)	.041	.037	.052	.040	.589	.186	.298	.015	.002	.910	.002	.215	.079	.007
Activity	All (n=20)	P3_A	Pearson correlation coeficient	.298	.291	.274	.291	,446*	.381	.440	.190	-.148	.246	-.139	-.008	.270	.089
			Sig. (bilateral)	.203	.213	.243	.214	.049	.097	.052	.422	.533	.295	.557	.973	.249	.709

RHYTHMIC VARIABLE		Physical_functioning	Physical_role_functioning	Bodily_pain	General_health_perception	Vitality	Social_role_functioning	Emocional_role_functioning	Mental_health	All _SF36	C1	C2	C3	C4	C5	C6	C7	Global_PSQI_score	number of significant pairs of correlation (p<0.05)	p real level of significance
mean 24h	Pearson correlation coeficient	,607**	,632**	,648**	,646**	,743**	,604**	.157	,483*	,668**	-.439	-,458*	-.269	-.419	-,518*	-,513*	-.433	-,642**		
	Sig. (bilateral)	.005	.003	.002	.002	.000	.005	.509	.031	.001	.053	.042	.251	.066	.019	.021	.056	.002	21	0.063
A_cos	Pearson correlation coeficient	.318	.256	.318	.351	.425	.244	-.077	.375	.313	-.188	-.148	-.198	-.137	-.240	-.251	-.206	-.282		
	Sig. (bilateral)	.171	.275	.172	.129	.062	.300	.746	.104	.180	.428	.532	.403	.564	.307	.286	.383	.229	1	0.0017
R_cos	Pearson correlation coeficient	-,579**	-,748**	-,631**	-,550*	-,682**	-,720**	-.327	-.328	-,695**	,483*	,566**	.137	,515*	,497*	,642**	,476*	,719**		
	Sig. (bilateral)	.008	.000	.003	.012	.001	.000	.159	.158	.001	.031	.009	.564	.020	.026	.002	.034	.000	26	0.069
RA_np	Pearson correlation coeficient	.174	.208	.151	.112	.238	.160	-.112	.197	.164	-.368	-.160	-.214	-.278	-.369	-.015	.274	-.218		
	Sig. (bilateral)	.464	.378	.526	.639	.313	.500	.638	.406	.490	.110	.501	.365	.236	.110	.951	.243	.356	1	0.0017
IV	Pearson correlation coeficient	.289	.394	.284	.116	.329	.417	.345	.177	.360	-.322	-.137	-.348	-.186	-.188	-.222	-.319	-.356		
	Sig. (bilateral)	.216	.085	.224	.626	.156	.067	.136	.455	.119	.167	.564	.132	.431	.427	.346	.171	.123	0	
R	Pearson correlation coeficient	.428	.275	.297	.276	.439	.341	.045	.223	.345	-.252	-.177	-,546*	-.238	-.105	.123	-.097	-.247		
	Sig. (bilateral)	.060	.240	.204	.239	.053	.141	.852	.344	.136	.284	.456	.013	.312	.661	.604	.684	.293	1	0.0017
PV	Pearson correlation coeficient	.288	.440	.350	.102	.321	.354	-.002	.202	.317	-.317	-.199	-.298	-.252	-.261	-.165	.106	-.282		
	Sig. (bilateral)	.218	.052	.131	.668	.167	.126	.992	.393	.174	.173	.400	.201	.284	.266	.486	.656	.229	0	
M10	Pearson correlation coeficient	,503*	,475*	,509*	,539*	,609**	.439	.048	.432	,520*	-.316	-.324	-.235	-.297	-.407	-.405	-.349	-,488*		
	Sig. (bilateral)	.024	.034	.022	.014	.004	.053	.840	.057	.019	.175	.163	.319	.203	.075	.077	.132	.029	13	0.0497
L5	Pearson correlation coeficient	.153	.105	.180	.241	.175	.141	.153	.065	.180	.126	-.079	.071	.093	.100	-.223	-,531*	-.111		
	Sig. (bilateral)	.519	.661	.448	.306	.460	.554	.520	.785	.448	.597	.741	.767	.698	.674	.344	.016	.642	1	0.0017
P1_A	Pearson correlation coeficient	.053	-.238	-.045	-.020	-.019	-.137	-.074	.164	-.070	.055	.151	.002	.150	.083	.192	.231	.194		
	Sig. (bilateral)	.824	.311	.852	.932	.938	.564	.755	.490	.769	.818	.526	.994	.527	.729	.417	.327	.413	0	
P2_A	Pearson correlation coeficient	,520*	,553*	.418	.272	.426	,546*	.171	-.013	,466*	-.351	-.367	-.407	-,709**	-,493*	-.329	-.163	-,592**		
	Sig. (bilateral)	.019	.011	.067	.246	.061	.013	.470	.956	.039	.129	.112	.075	.000	.027	.156	.491	.006	14	0.0517
P3_A	Pearson correlation coeficient	-.055	-.323	-.190	-.265	-.297	-.177	-.226	-,471*	-.274	,448*	.034	-.254	-.310	.108	,488*	.272	.184		
	Sig. (bilateral)	.819	.165	.422	.260	.203	.456	.337	.036	.242	.048	.888	.279	.183	.650	.029	.246	.438	4	0.02

Season: Winter. Variable: Distal Skin Temperature. Subjects: All

VARIABLE (Activity or Distal Skin Temperatureerature)	SUBJECTS	RHYTHMIC VARIABLE		Scorefis40_1	Scorefis40_2	Scorefis40_3	All _fis40	ScoreHADS_1	ScoreHADS_2	All _HADS	C31_OI	C31_vasom	C31_secremot	C31_GI	C31_Bladder	C31_pupilmot	C31_All
Distal Skin Temperature	All (n=20)	mean 24h	Pearson correlation coeficient	.063	.039	.069	.053	.198	.194	.210	.060	0.11552	.136	-.086	-.006	.069	.032
			Sig. (bilateral)	.790	.871	.772	.823	.402	.413	.375	.802	0.62767	.568	.719	.982	.771	.892
Distal Skin Temperature	All (n=20)	A_cos	Pearson correlation coeficient	-.069	-.061	-.107	-.076	.090	.097	.101	.079	-0.0695	.106	.111	-.005	.090	.087
			Sig. (bilateral)	.774	.798	.655	.751	.707	.683	.672	.742	0.77086	.657	.643	.983	.705	.715
Distal Skin Temperature	All (n=20)	RA_cos	Pearson correlation coeficient	-.053	-.043	-.093	-.059	.082	.088	.092	.093	-0.0513	.104	.131	.015	.096	.104
			Sig. (bilateral)	.825	.858	.695	.804	.731	.712	.701	.696	0.82983	.662	.582	.949	.686	.664
Distal Skin Temperature	All (n=20)	RA_np	Pearson correlation coeficient	-.179	-.171	-.235	-.192	.021	.030	.028	-.038	-0.1354	.039	.016	-.123	-.035	-.039
			Sig. (bilateral)	.449	.470	.318	.417	.931	.899	.907	.873	0.5693	.872	.946	.606	.884	.869
Distal Skin Temperature	All (n=20)	IV	Pearson correlation coeficient	.034	.065	.000	.040	.077	.246	.183	.062	0.09248	.085	.030	-.206	.138	.063
			Sig. (bilateral)	.886	.786	.999	.866	.748	.295	.439	.796	0.69818	.721	.901	.383	.561	.790
Distal Skin Temperature	All (n=20)	R	Pearson correlation coeficient	-.306	-.367	-.328	-.345	-.219	-.346	-.310	-.325	-,522*	.055	-.346	.060	-.328	-.343
			Sig. (bilateral)	.189	.112	.158	.137	.354	.135	.183	.161	0.01811	.817	.135	.803	.158	.139
Distal Skin Temperature	All (n=20)	PV	Pearson correlation coeficient	-.112	-.144	-.215	-.156	.172	.129	.159	-.065	-0.2912	.166	-.266	-.146	-.107	-.172
			Sig. (bilateral)	.639	.546	.363	.511	.469	.587	.504	.786	0.21297	.485	.258	.539	.652	.469
Distal Skin Temperature	All (n=20)	M5	Pearson correlation coeficient	-.392	-.410	-,476*	-.427	.108	.110	.117	-.229	-0.2351	.090	-.297	-.400	-.221	-.287
			Sig. (bilateral)	.087	.072	.034	.060	.652	.644	.624	.331	0.31838	.707	.204	.080	.349	.220
Distal Skin Temperature	All (n=20)	L10	Pearson correlation coeficient	.022	.005	.039	.018	.045	.035	.042	-.050	0.05795	.015	-.151	-.048	-.055	-.078
			Sig. (bilateral)	.926	.985	.870	.939	.851	.884	.860	.833	0.80825	.950	.526	.841	.816	.743
Distal Skin Temperature	All (n=20)	P1_T	Pearson correlation coeficient	.166	.138	.097	.135	.313	.320	.340	.245	-.001	.177	.061	.022	.217	.166
			Sig. (bilateral)	.483	.562	.683	.569	.178	.169	.142	.299	.998	.456	.800	.928	.357	.484
Distal Skin Temperature	All (n=20)	P2_T	Pearson correlation coeficient	-.428	-.423	-,486*	-,445*	-.188	-.276	-.254	-,463*	-,461*	-.133	-,586**	-.324	-,561**	-,586**
			Sig. (bilateral)	.060	.063	.030	.049	.426	.239	.279	.040	.041	.576	.007	.163	.010	.007
Distal Skin Temperature	All (n=20)	P3_T	Pearson correlation coeficient	.375	.384	.339	.373	.017	.117	.078	.387	.212	.443	.396	,555*	.365	,472*
			Sig. (bilateral)	.103	.095	.144	.105	.943	.622	.744	.092	.369	.050	.084	.011	.114	.036

RHYTHMIC VARIABLE		Physical_functioning	Physical_role_functioning	Bodily_pain	General_health_perception	Vitality	Social_role_functioning	Emocional_role_functioning	Mental_health	All _SF36	C1	C2	C3	C4	C5	C6	C7	Global_PSQI_score	number of significant pairs of correlation (p<0.05)	p real level of significance
mean 24h	Pearson correlation coeficient	.020	-.035	-.214	-0.2881	.011	.057	-,458*	-.216	-.147	.206	.078	-.164	-.140	.034	-0.0793	-.359	-.092		
	Sig. (bilateral)	.933	.884	.364	.218	.964	.812	.042	.361	.536	.384	.744	.489	.555	.888	0.73958	.120	.699	1	0.0017
A_cos	Pearson correlation coeficient	.163	.116	.350	0.27609	-.047	.039	,472*	-.046	.207	-.032	-.172	-.133	-.101	-.269	0.24573	.206	-.020		
	Sig. (bilateral)	.492	.627	.131	.239	.845	.871	.036	.847	.382	.894	.470	.576	.671	.252	0.29634	.382	.932	1	0.0017
RA_cos	Pearson correlation coeficient	.139	.098	.327	0.26981	-.056	.022	,461*	-.034	.190	-.041	-.144	-.119	-.075	-.244	0.25681	.221	.002		
	Sig. (bilateral)	.559	.682	.160	.250	.815	.928	.041	.887	.422	.865	.544	.616	.754	.299	0.27439	.349	.994	1	0.0017
RA_np	Pearson correlation coeficient	.198	.238	,450*	0.36382	.080	.163	,493*	.015	.308	-.134	-.271	-.151	-.205	-.389	0.16572	.175	-.138		
	Sig. (bilateral)	.404	.313	.047	.115	.739	.492	.027	.951	.187	.573	.248	.526	.387	.090	0.48502	.462	.563	2	0.0081
IV	Pearson correlation coeficient	-.347	.133	-.085	-0.0296	.009	.042	-.394	-.053	-.105	-.057	.019	.165	-.041	-.088	0.05208	.053	.021		
	Sig. (bilateral)	.134	.575	.723	.901	.971	.862	.085	.823	.659	.813	.935	.487	.863	.711	0.82739	.823	.932	0	
R	Pearson correlation coeficient	.376	.368	,605**	0.2554	.133	.364	,584**	.086	.432	-.027	-.297	.002	-.321	-.215	-0.3554	.093	-.254		
	Sig. (bilateral)	.102	.111	.005	.277	.575	.115	.007	.720	.057	.909	.203	.994	.167	.363	0.12415	.696	.281	3	0.0147
PV	Pearson correlation coeficient	.302	.219	,475*	0.16138	.096	.299	.394	-.136	.297	-.076	-.344	-.374	-.438	-,498*	0.09513	.209	-.262		
	Sig. (bilateral)	.195	.354	.034	.497	.686	.200	.086	.569	.204	.751	.138	.104	.054	.025	0.68992	.376	.265	2	0.008
M5	Pearson correlation coeficient	.409	,488*	,568**	0.32879	.348	,496*	.230	-.087	,449*	-.106	-,497*	-.427	-,633**	-,680**	-0.0219	-.260	-,515*		
	Sig. (bilateral)	.073	.029	.009	.157	.133	.026	.329	.717	.047	.656	.026	.060	.003	.001	0.92684	.268	.020	9	0.0399
L10	Pearson correlation coeficient	-.030	-.037	-.243	-0.261	.076	.056	-,464*	-.067	-.138	.104	.077	-.050	-.077	.107	-0.1823	-.331	-.091		
	Sig. (bilateral)	.901	.878	.303	.266	.751	.816	.039	.777	.561	.663	.746	.836	.745	.653	0.44184	.154	.703	1	0.0017
P1_T	Pearson correlation coeficient	.087	-.132	.203	.083	-.211	-.070	.322	-.249	.020	.224	-.119	-.223	-.130	-.157	.424	.269	.100		
	Sig. (bilateral)	.714	.579	.391	.728	.371	.768	.166	.289	.932	.341	.618	.344	.584	.509	.063	.252	.674	0	
P2_T	Pearson correlation coeficient	.329	,483*	,481*	.288	.407	,459*	.211	.113	.429	-.373	-.408	-.237	-,583**	-,582**	-.413	-.123	-,570**		
	Sig. (bilateral)	.157	.031	.032	.219	.075	.042	.373	.636	.059	.105	.074	.315	.007	.007	.070	.607	.009	13	0.0497
P3_T	Pearson correlation coeficient	-.278	-.267	-.257	-.382	-.300	-.147	-.051	.042	-.258	.022	.369	.187	,488*	.327	.074	.441	.398		
	Sig. (bilateral)	.236	.255	.273	.097	.199	.538	.831	.861	.272	.926	.109	.429	.029	.160	.757	.052	.082	3	0.0147

Season: Summer. Variable: activity. Subjects: All

VARIABLE (Activity or Distal Skin Temperatureerature)	SUBJECTS	RHYTHMIC VARIABLE		Scorefis40_1	Scorefis40_2	Scorefis40_3	All _fis40	ScoreHADS_1	ScoreHADS_2	All _HADS	C31_OI	C31_vasom	C31_secremot	C31_GI	C31_Bladder	C31_pupilmot	C31_All
Activity	ALL (N=18)	mean 24h	Pearson correlation coeficient	-,498*	-,490*	-,548*	-,512*	-.164	-.453	-.329	-.287	-0.1026	-.269	-.268	-.215	-.376	-.386
			Sig. (bilateral)	.035	.039	.019	.030	.516	.059	.183	.247	0.68534	.280	.282	.391	.124	.125
Activity	ALL (N=18)	A_cos	Pearson correlation coeficient	-.160	-.159	-.236	-.182	.125	-.189	-.041	.007	0.14369	-.019	.045	-.075	.014	.005
			Sig. (bilateral)	.525	.529	.345	.471	.620	.452	.872	.979	0.56946	.941	.859	.768	.955	.984
Activity	ALL (N=18)	R_cos	Pearson correlation coeficient	,523*	,514*	,499*	,517*	.437	.457	.467	.428	0.36562	.373	.458	.188	,588*	,607**
			Sig. (bilateral)	.026	.029	.035	.028	.070	.056	.051	.076	0.13568	.128	.056	.455	.010	.010
Activity	ALL (N=18)	RA_np	Pearson correlation coeficient	.045	.063	.065	.060	.220	.001	.110	.008	0.18903	.026	.299	.061	.321	.337
			Sig. (bilateral)	.861	.803	.796	.814	.381	.998	.665	.976	0.45251	.917	.228	.809	.194	.185
Activity	ALL (N=18)	IV	Pearson correlation coeficient	-.452	-.434	-.349	-.419	-,543*	-.333	-.452	-.394	-0.424	-.366	-.425	-.233	-,544*	-.443
			Sig. (bilateral)	.060	.072	.156	.083	.020	.177	.060	.106	0.07954	.136	.078	.353	.020	.075
Activity	ALL (N=18)	R	Pearson correlation coeficient	-.117	-.089	-.117	-.104	.005	.077	.045	-.310	-0.1498	-.057	-.181	-.055	-.082	-.116
			Sig. (bilateral)	.643	.726	.643	.681	.984	.761	.861	.210	0.55307	.823	.471	.829	.747	.659
Activity	ALL (N=18)	PV	Pearson correlation coeficient	-.380	-.361	-.343	-.364	-.191	-.227	-.219	-.411	-0.2026	-.320	-.406	-.242	-.216	-.293
			Sig. (bilateral)	.119	.141	.163	.138	.448	.364	.382	.091	0.41999	.196	.095	.332	.389	.254
Activity	ALL (N=18)	M10	Pearson correlation coeficient	-.352	-.337	-.398	-.360	-.028	-.347	-.204	-.137	0.03884	-.172	-.116	-.160	-.171	-.191
			Sig. (bilateral)	.152	.171	.102	.142	.912	.158	.418	.587	0.87838	.495	.645	.526	.497	.462
Activity	ALL (N=18)	L5	Pearson correlation coeficient	-.231	-.238	-.254	-.243	-.282	-.179	-.238	-.104	-0.1833	-.113	-.383	-.108	-.430	-.444
			Sig. (bilateral)	.356	.341	.309	.331	.256	.478	.341	.681	0.46656	.654	.117	.669	.075	.074
Activity	ALL (N=18)	P1_A	Pearson correlation coeficient	.047	.027	-.113	-.005	.176	.065	.123	-.009	0.03545	.063	-.031	-.005	.013	-.036
			Sig. (bilateral)	.852	.914	.656	.983	.486	.799	.628	.972	0.88894	.803	.904	.983	.959	.890
Activity	ALL (N=18)	P2_A	Pearson correlation coeficient	-.434	-.428	-.409	-.428	-.256	-.329	-.307	-.409	-0.3185	-.297	-.395	-.175	-.322	-.376
			Sig. (bilateral)	.072	.076	.092	.077	.306	.182	.215	.092	0.19775	.231	.105	.488	.193	.137
Activity	ALL (N=18)	P3_A	Pearson correlation coeficient	.217	.244	.280	.249	.324	.284	.316	.117	-0.149	-.046	.028	-.064	.285	.234
			Sig. (bilateral)	.388	.330	.260	.319	.190	.254	.202	.644	0.55521	.856	.914	.800	.252	.365

RHYTHMIC VARIABLE		Physical_functioning	Physical_role_functioning	Bodily_pain	General_health_perception	Vitality	Social_role_functioning	Emocional_role_functioning	Mental_health	All _SF36	C1	C2	C3	C4	C5	C6	C7	Global_PSQI_score		
mean 24h	Pearson correlation coeficient	,475*	.443	,484*	0.40918	,539*	.334	-.042	.242	,550*	-.197	-.211	.281	-.008	-.307	-0.3633	-.305	-.196		
	Sig. (bilateral)	.046	.066	.042	.092	.021	.176	.870	.334	.018	.434	.417	.274	.976	.215	0.13843	.219	.436	8	0.0368
A_cos	Pearson correlation coeficient	.147	.172	.158	0.13399	.291	.065	-.297	-.042	.142	-.026	.090	.371	.376	-.036	-0.1342	-.096	.109		
	Sig. (bilateral)	.562	.496	.531	.596	.242	.799	.232	.870	.575	.919	.732	.143	.137	.887	0.59539	.703	.667	0	
R_cos	Pearson correlation coeficient	-,507*	-.415	-,510*	-0.4091	-.405	-.413	-.339	-.435	-,656**	.254	.446	.134	,567*	.425	0.4094	.283	.445		
	Sig. (bilateral)	.032	.087	.030	.092	.096	.088	.168	.071	.003	.309	.072	.609	.018	.079	0.09158	.255	.064	10	0.0427
RA_np	Pearson correlation coeficient	-.076	.012	-.121	0.01789	.052	-.173	-.359	-.083	-.319	.101	.392	.273	,494*	.229	0.05159	-.107	.192		
	Sig. (bilateral)	.765	.962	.633	.944	.838	.492	.144	.744	.198	.691	.119	.290	.044	.361	0.83889	.673	.445	1	0.0017
IV	Pearson correlation coeficient	.263	.333	.327	0.41461	.232	,470*	,597**	.428	.273	-,552*	-,593*	-.333	-,536*	-,493*	-0.2025	-.353	-,626**		
	Sig. (bilateral)	.291	.177	.185	.087	.355	.049	.009	.076	.274	.018	.012	.192	.027	.038	0.4203	.150	.005	9	0.0399
R	Pearson correlation coeficient	.047	.124	.134	0.17399	.094	.134	-.056	.089	-.044	-.031	-.388	.310	.172	-.033	0.30353	-.215	-.013		
	Sig. (bilateral)	.853	.624	.595	.490	.710	.595	.826	.725	.861	.904	.124	.227	.509	.897	0.22078	.390	.960	0	
PV	Pearson correlation coeficient	.352	.412	.364	0.40111	.446	.292	-.023	.220	.046	-.325	-.326	.373	.005	-.174	-0.142	-.359	-.291		
	Sig. (bilateral)	.152	.089	.138	.099	.064	.240	.928	.380	.856	.188	.201	.140	.984	.489	0.5741	.143	.241	0	
M10	Pearson correlation coeficient	.333	.317	.335	0.29192	.421	.186	-.183	.097	.358	-.109	-.022	.351	.203	-.190	-0.29	-.194	-.038		
	Sig. (bilateral)	.177	.199	.175	.240	.081	.460	.467	.701	.145	.667	.932	.167	.435	.450	0.24302	.441	.879	0	
L5	Pearson correlation coeficient	.243	.156	.269	0.14705	.149	.276	.319	.186	,468*	-.184	-.438	-.147	-.447	-.283	-0.1792	-.047	-.258		
	Sig. (bilateral)	.331	.536	.281	.560	.554	.268	.197	.459	.050	.465	.079	.574	.072	.255	0.47681	.852	.301	1	0.0017
P1_A	Pearson correlation coeficient	-.040	.039	.051	-0.0389	.153	.044	-.304	-.066	.077	.056	-.110	.259	.291	.188	0.18868	.035	.203		
	Sig. (bilateral)	.873	.879	.841	.878	.543	.861	.221	.795	.762	.826	.673	.315	.257	.455	0.45337	.890	.419	0	
P2_A	Pearson correlation coeficient	.391	.362	.428	0.42045	.407	.271	.041	.311	.336	-.248	-.296	.062	-.336	-.127	-0.115	-.312	-.293		
	Sig. (bilateral)	.109	.140	.076	.082	.094	.276	.873	.210	.173	.321	.249	.812	.187	.615	0.6496	.207	.238	0	
P3_A	Pearson correlation coeficient	-.171	-.137	-.208	-0.1626	-.229	-.182	-.347	-.368	-,525*	.035	.318	.023	.113	.260	-0.0809	.069	.034		
	Sig. (bilateral)	.497	.587	.408	.519	.361	.469	.159	.133	.025	.891	.214	.929	.666	.298	0.74957	.784	.894	1	0.0017

Season: Summer. Variable: Distal Skin Temperature. m Subjects: All

VARIABLE (Activity or Distal Skin Temperatureerature)	SUBJECTS	RHYTHMIC VARIABLE		Scorefis40_1	Scorefis40_2	Scorefis40_3	All _fis40	ScoreHADS_1	ScoreHADS_2	All _HADS	C31_OI	C31_vasom	C31_secremot	C31_GI	C31_Bladder	C31_pupilmot	C31_All
Distal Skin Temperature	ALL (N=18)	mean 24h	Pearson correlation coeficient	.360	.323	.212	.305	.256	.442	.369	.412	0.21675	.309	.217	.015	.094	.106
			Sig. (bilateral)	.142	.191	.398	.219	.306	.066	.132	.089	0.38763	.212	.387	.953	.710	.685
Distal Skin Temperature	ALL (N=18)	A_cos	Pearson correlation coeficient	-.192	-.174	-.074	-.153	-.111	-.279	-.208	-.224	-0.0647	-.236	-.037	.065	.025	.196
			Sig. (bilateral)	.445	.490	.770	.544	.660	.262	.408	.371	0.79874	.346	.884	.799	.921	.452
Distal Skin Temperature	ALL (N=18)	RA_cos	Pearson correlation coeficient	-.203	-.186	-.082	-.164	-.119	-.274	-.209	-.242	-0.083	-.246	-.060	.038	.019	.192
			Sig. (bilateral)	.418	.461	.746	.517	.639	.271	.405	.333	0.74345	.325	.814	.882	.939	.460
Distal Skin Temperature	ALL (N=18)	RA_np	Pearson correlation coeficient	-.273	-.247	-.145	-.228	-.157	-.332	-.259	-.303	-0.123	-.299	-.105	.011	-.037	.063
			Sig. (bilateral)	.274	.322	.567	.363	.535	.178	.299	.221	0.62673	.229	.678	.967	.885	.811
Distal Skin Temperature	ALL (N=18)	IV	Pearson correlation coeficient	.272	.235	.182	.232	.146	.260	.215	.209	0.02997	.230	.089	-.021	.167	.070
			Sig. (bilateral)	.276	.347	.471	.355	.563	.297	.392	.405	0.90602	.359	.726	.935	.509	.788
Distal Skin Temperature	ALL (N=18)	R	Pearson correlation coeficient	.153	.175	.238	.188	-.210	-.154	-.189	.158	0.14279	.123	.201	.423	.214	.285
			Sig. (bilateral)	.545	.487	.342	.455	.402	.541	.453	.532	0.57191	.626	.425	.080	.394	.268
Distal Skin Temperature	ALL (N=18)	PV	Pearson correlation coeficient	-.055	-.033	.064	-.013	-.287	-.312	-.313	-.044	0.09627	.024	.110	.314	.098	.264
			Sig. (bilateral)	.827	.896	.802	.960	.248	.207	.205	.864	0.70395	.925	.664	.205	.699	.307
Distal Skin Temperature	ALL (N=18)	M5	Pearson correlation coeficient	.140	.132	.064	.117	.204	.200	.211	.234	0.19337	.066	.214	.087	.025	.068
			Sig. (bilateral)	.578	.601	.802	.644	.417	.426	.401	.350	0.44202	.794	.394	.731	.923	.795
Distal Skin Temperature	ALL (N=18)	L10	Pearson correlation coeficient	.292	.265	.154	.244	.203	.378	.307	.340	0.16101	.289	.146	-.007	.044	-.029
			Sig. (bilateral)	.240	.289	.541	.330	.420	.122	.215	.167	0.5233	.245	.563	.977	.861	.913
Distal Skin Temperature	ALL (N=18)	P1_T	Pearson correlation coeficient	.071	.082	.174	.105	-.052	-.147	-.106	.029	0.10445	.009	.187	.294	.220	.467
			Sig. (bilateral)	.778	.746	.489	.678	.838	.561	.675	.908	0.67999	.973	.457	.236	.379	.059
Distal Skin Temperature	ALL (N=18)	P2_T	Pearson correlation coeficient	-.317	-.278	-.206	-.270	-,521*	-,502*	-,533*	-.136	0.1081	.056	-.054	.225	-.146	-.083
			Sig. (bilateral)	.200	.264	.413	.278	.027	.034	.023	.592	0.6694	.827	.832	.370	.564	.752
Distal Skin Temperature	ALL (N=18)	P3_T	Pearson correlation coeficient	.287	.275	.227	.267	-.082	.150	.041	.185	-0.0544	.316	.153	.216	.022	.124
			Sig. (bilateral)	.249	.270	.365	.284	.746	.554	.872	.463	0.83031	.201	.543	.389	.932	.637

RHYTHMIC VARIABLE		Physical_functioning	Physical_role_functioning	Bodily_pain	General_health_perception	Vitality	Social_role_functioning	Emocional_role_functioning	Mental_health	All _SF36	C1	C2	C3	C4	C5	C6	C7	Global_PSQI_score		
mean 24h	Pearson correlation coeficient	-.292	-.360	-.191	-0.3912	-.305	-.196	-.150	-.339	.156	.341	-.190	.277	.036	.059	0.22815	.344	.347		
	Sig. (bilateral)	.241	.143	.448	.108	.219	.436	.551	.168	.537	.167	.465	.283	.892	.817	0.36253	.162	.159	0	
A_cos	Pearson correlation coeficient	.177	.236	.075	0.28082	.161	.133	.220	.106	-.262	-.315	.458	-.313	.132	-.113	-0.1652	-.109	-.238		
	Sig. (bilateral)	.482	.346	.768	.259	.524	.598	.379	.676	.294	.203	.064	.221	.613	.654	0.51246	.668	.342	0	
RA_cos	Pearson correlation coeficient	.190	.249	.087	0.28998	.175	.143	.211	.118	-.268	-.320	.452	-.313	.132	-.122	-0.1659	-.131	-.254		
	Sig. (bilateral)	.451	.319	.730	.243	.487	.571	.401	.640	.281	.195	.068	.221	.613	.630	0.51049	.604	.309	0	
RA_np	Pearson correlation coeficient	.245	.304	.154	0.36254	.215	.189	.234	.161	-.201	-.340	.318	-.283	.085	-.162	-0.174	-.171	-.285		
	Sig. (bilateral)	.328	.221	.543	.139	.392	.452	.350	.524	.423	.167	.214	.271	.745	.522	0.48987	.498	.251	0	
IV	Pearson correlation coeficient	-.198	-.265	-.089	-0.2883	-.182	-.131	-.182	-.237	-.008	.180	-.082	.212	.033	-.055	0.06914	.346	.201		
	Sig. (bilateral)	.432	.287	.725	.246	.469	.605	.469	.344	.975	.475	.754	.414	.901	.830	0.78515	.160	.424	0	
R	Pearson correlation coeficient	-.289	-.235	-.244	-0.1272	-.208	-.161	.174	.044	-.279	-.181	.279	-.211	.026	.047	-0.064	.283	.008		
	Sig. (bilateral)	.245	.349	.328	.615	.407	.523	.490	.863	.262	.473	.278	.415	.920	.852	0.80073	.255	.974	0	
PV	Pearson correlation coeficient	-.066	.005	-.079	0.13652	-.023	.018	.301	.171	-.247	-.307	.210	-.211	.065	-.070	-0.0885	.027	-.155		
	Sig. (bilateral)	.794	.986	.755	.589	.928	.942	.224	.498	.323	.215	.420	.415	.803	.782	0.72708	.916	.539	0	
M5	Pearson correlation coeficient	-.118	-.155	-.014	-0.0466	-.223	-.031	.123	-.349	.148	.130	-.006	.064	.128	-.170	0.24997	.348	.243		
	Sig. (bilateral)	.641	.540	.956	.854	.373	.901	.626	.156	.557	.607	.981	.807	.625	.500	0.31712	.158	.332	0	
L10	Pearson correlation coeficient	-.255	-.319	-.143	-0.3472	-.256	-.180	-.186	-.248	.212	.347	-.360	.356	-.023	.091	0.23291	.249	.322		
	Sig. (bilateral)	.308	.196	.572	.158	.305	.475	.460	.321	.398	.159	.156	.161	.931	.720	0.35232	.320	.192	0	
P1_T	Pearson correlation coeficient	-.110	-.040	-.174	0.02666	-.101	-.058	.206	-.027	-.430	-.204	,541*	-.313	.227	.014	-0.0516	.120	-.063		
	Sig. (bilateral)	.665	.873	.490	.916	.691	.820	.412	.917	.075	.417	.025	.221	.381	.958	0.83899	.635	.804	1	0.0017
P2_T	Pearson correlation coeficient	.106	.091	.147	0.29452	.143	.039	.216	,526*	.284	-.129	-.206	-.018	-.143	-.047	0.04511	-.260	-.123		
	Sig. (bilateral)	.674	.720	.561	.235	.571	.878	.388	.025	.254	.611	.427	.945	.585	.854	0.85893	.298	.628	4	0.0204
P3_T	Pearson correlation coeficient	-.325	-.254	-.280	-0.1956	-.305	.076	.144	-.081	-.106	-.224	-.241	-.255	-.104	.095	-0.0781	.201	-.097		
	Sig. (bilateral)	.188	.308	.260	.437	.219	.765	.568	.750	.674	.371	.352	.323	.691	.708	0.75801	.424	.701	0	

Season: Summer and winter. Variable: activity. Subjects: All

VARIABLE (Activity or Distal Skin Temperatureerature)	SUBJECTS	RHYTHMIC VARIABLE		Scorefis40_1	Scorefis40_2	Scorefis40_3	All _fis40	ScoreHADS_1	ScoreHADS_2	All _HADS	C31_OI	C31_vasom	C31_secremot	C31_GI	C31_Bladder	C31_pupilmot	C31_All	Physical_functioning	Physical_role_functioning	Bodily_pain	General_health_perception	Vitality	Social_role_functioning	Emocional_role_functioning	Mental_health	All _SF36	C1	C2	C3	C4	C5	C6	C7	Global_PSQI_score		
Activity	ALL (N=38)	mean 24h	Pearson correlation coeficient	-,407*	-,546**	-,534**	-,455**	-.177	-,437**	-,335*	-,453**	-0.1483	-.235	-,387*	-,422**	-,470**	-,449**	,542**	,554**	,567**	,540**	,626**	,492**	.065	,364*	,550**	-,355*	-,364*	-.013	-.255	-,415**	-,449**	-,369*	-,443**		
			Sig. (bilateral)	.011	.000	.001	.004	.289	.006	.040	.004	0.37422	.155	.016	.008	.003	.005	.000	.000	.000	.000	.000	.002	.698	.025	.000	.029	.027	.941	.127	.010	0.00467	.023	.005	25	.068
Activity	ALL (N=38)	A_cos	Pearson correlation coeficient	-.232	-.214	-.269	-.178	.080	-.164	-.055	-.141	0.15119	-.084	-.102	-.294	-.126	-.127	.242	.227	.249	0.25986	,350*	.175	-.170	.187	.219	-.141	-.069	.074	.063	-.149	-0.2065	-.152	-.113		
			Sig. (bilateral)	.162	.197	.102	.284	.634	.325	.745	.397	0.36488	.618	.544	.073	.451	.446	.144	.171	.132	.115	.031	.294	.308	.262	.187	.397	.684	.663	.711	.373	0.21362	.362	.500	1	0.0017
Activity	ALL (N=38)	R_cos	Pearson correlation coeficient	,325*	,619**	,481**	,520**	,448**	,513**	,511**	,570**	,481**	.285	,514**	.266	,611**	,579**	-,545**	-,591**	-,565**	-,481**	-,531**	-,578**	-,334*	-,377*	-,590**	,376*	,499**	.156	,541**	,459**	,529**	,387*	,590**		
			Sig. (bilateral)	.047	.000	.002	.001	.005	.001	.001	.000	0.00221	.082	.001	.106	.000	.000	.000	.000	.000	.002	.001	.000	.041	.020	.000	.020	.002	.358	.001	.004	0.00064	.016	.000	28	.070
Activity	ALL (N=38)	RA_np	Pearson correlation coeficient	-.047	-.003	-.046	-.004	.184	.016	.099	-.120	0.0156	-.003	.077	-.182	.172	.035	.026	.098	-.010	0.05756	.120	-.028	-.260	.024	.004	-.115	.123	.110	.140	-.003	0.01852	.053	.028		
			Sig. (bilateral)	.777	.984	.783	.979	.268	.926	.556	.472	0.92593	.987	.646	.273	.301	.832	.878	.558	.953	.731	.472	.867	.115	.884	.979	.491	.469	.518	.409	.984	0.91212	.751	.867	0	
Activity	ALL (N=38)	IV	Pearson correlation coeficient	-,355*	-.309	-,409*	-.181	-,426**	-,356*	-,411*	-.261	-0.2885	-.107	-.183	-.036	-,401*	-.278	.270	,372*	.305	0.24608	.275	,436**	,440**	.295	,385*	-,422**	-,334*	-.250	-.291	-.320	-0.2183	-.319	-,454**		
			Sig. (bilateral)	.029	.059	.011	.278	.008	.028	.010	.113	0.07899	.521	.270	.831	.013	.091	.102	.022	.063	.136	.095	.006	.006	.072	.017	.008	.043	.135	.081	.050	0.188	.051	.004	13	0.0497
Activity	ALL (N=38)	R	Pearson correlation coeficient	-.153	-.233	-.191	-.180	.064	-.063	-.005	-.233	-0.1152	-.051	-.151	-.231	-.087	-.165	.253	.211	.221	0.22961	.261	.251	-.006	.157	.230	-.166	-.276	-.113	-.058	-.070	0.20004	-.147	-.134		
			Sig. (bilateral)	.358	.159	.251	.279	.702	.706	.977	.160	0.49115	.763	.365	.163	.605	.321	.126	.202	.183	.166	.113	.129	.970	.346	.164	.318	.098	.506	.734	.677	0.22853	.379	.421	0	
Activity	ALL (N=38)	PV	Pearson correlation coeficient	-.280	-.263	-,322*	-.181	-.093	-.155	-.134	-,327*	-0.2353	-.154	-,348*	-.266	-.153	-.299	.318	,416**	,356*	0.27319	,399*	.313	-.016	.214	,337*	-.313	-.272	.099	-.108	-.207	-0.1508	-.158	-.281		
			Sig. (bilateral)	.089	.111	.049	.276	.579	.353	.424	.045	0.15499	.357	.032	.107	.359	.068	.052	.009	.028	.097	.013	.056	.925	.197	.039	.056	.103	.562	.523	.212	0.36605	.343	.088	7	0.0335
Activity	ALL (N=38)	M10	Pearson correlation coeficient	-,329*	-,406*	-,417**	-,339*	-.051	-.318	-.206	-.311	0.00816	-.178	-.253	-,376*	-.299	-.298	,424**	,412*	,427**	,431**	,504**	,336*	-.054	.275	,403*	-.248	-.213	.041	-.100	-.305	-,357*	-.275	-.290		
			Sig. (bilateral)	.044	.011	.009	.037	.761	.052	.214	.057	0.96124	.285	.125	.020	.068	.069	.008	.010	.007	.007	.001	.039	.745	.095	.012	.133	.205	.812	.557	.063	0.02779	.095	.078	13	0.0497
Activity	ALL (N=38)	L5	Pearson correlation coeficient	-.124	-.210	-.175	-.170	-.246	-.198	-.233	-.053	-0.041	-.106	-.240	.012	-,353*	-.213	.202	.126	.227	0.17419	.154	.211	.254	.139	.216	-.043	-.260	-.099	-.211	-.143	-0.1848	-.226	-.198		
			Sig. (bilateral)	.460	.206	.295	.309	.137	.234	.160	.751	0.80691	.528	.146	.941	.030	.199	.224	.452	.170	.296	.355	.203	.124	.404	.193	.795	.119	.558	.210	.393	0.2667	.172	.232	1	0.0017
Activity	ALL (N=38)	P1_A	Pearson correlation coeficient	-.041	.148	-.007	.111	.175	.077	.129	.049	0.15232	.036	.014	-.078	.025	.032	-.070	-.094	.013	-0.0437	.067	-.050	-.214	.132	-.052	.003	.012	.098	.212	.127	0.18366	.186	.189		
			Sig. (bilateral)	.808	.374	.965	.508	.294	.648	.441	.771	0.36127	.828	.936	.640	.881	.848	.677	.576	.939	.794	.691	.764	.198	.430	.756	.985	.942	.562	.208	.448	0.26969	.264	.256	0	
Activity	ALL (N=38)	P2_A	Pearson correlation coeficient	-,321*	-,462**	-,427**	-,370*	-.221	-,340*	-.302	-,497**	-,508**	-.129	-,539**	-.282	-,392*	-,494**	,468**	,504**	,457**	,382*	,480**	,437**	.103	.211	,455**	-,372*	-,371*	-.144	-,504**	-.317	-0.2347	-.276	-,468**		
			Sig. (bilateral)	.049	.004	.007	.022	.182	.037	.066	.001	0.00113	.442	.000	.087	.015	.002	.003	.001	.004	.018	.002	.006	.540	.204	.004	.022	.024	.397	.001	.053	0.15606	.093	.003	21	0.063
Activity	ALL (N=38)	P3_A	Pearson correlation coeficient	.231	.187	.259	.135	,380*	,330*	,373*	.169	-0.1356	.034	-.019	-.039	.287	.100	-.111	-.233	-.202	-0.2066	-.260	-.182	-.284	-,429**	-.260	.258	.166	-.144	-.148	.188	0.20674	.162	.096		
			Sig. (bilateral)	.163	.261	.116	.418	.019	.043	.021	.311	0.41706	.839	.911	.814	.080	.552	.508	.159	.223	.213	.115	.273	.084	.007	.115	.118	.327	.396	.383	.258	0.213	.331	.566	4	0.02

Season: Summer and winter. Variable: Temperature. Subjects: All

VARIABLE (Activity or Distal Skin Temperatureerature)	SUBJECTS	RHYTHMIC VARIABLE		Scorefis40_1	Scorefis40_2	Scorefis40_3	All _fis40	ScoreHADS_1	ScoreHADS_2	All _HADS	C31_OI	C31_vasom	C31_secremot	C31_GI	C31_Bladder	C31_pupilmot	C31_All	Physical_functioning	Physical_role_functioning	Bodily_pain	General_health_perception	Vitality	Social_role_functioning	Emocional_role_functioning	Mental_health	All _SF36	C1	C2	C3	C4	C5	C6	C7	Global_PSQI_score		
Distal Skin Temperature	ALL (N=38)	mean 24h	Pearson correlation coeficient	,547**	-.123	,438**	-.310	.203	,331*	.287	.255	0.20482	.051	.171	.041	.137	.180	-.112	-.206	-.189	-0.2868	-.158	-.085	-.189	-.260	-.207	.277	.062	-.233	-.128	.043	0.10857	.011	.095		
			Sig. (bilateral)	.000	.463	.006	.058	.222	.043	.080	.122	0.21737	.761	.303	.806	.410	.279	.503	.214	.255	.081	.342	.610	.257	.115	.212	.092	.716	.165	.450	.798	0.51643	.946	.569	3	0.0147
Distal Skin Temperature	ALL (N=38)	A_cos	Pearson correlation coeficient	-,327*	.057	-.282	.171	-.031	-.130	-.089	-.100	-0.1056	-.004	-.029	.008	.006	-.037	.151	.184	.210	0.26806	.070	.090	.301	.053	.198	-.190	-.024	.019	.034	-.176	0.01714	.061	-.101		
			Sig. (bilateral)	.045	.734	.087	.306	.853	.437	.594	.552	0.52821	.980	.863	.960	.969	.823	.367	.268	.205	.104	.677	.590	.067	.751	.233	.253	.890	.909	.842	.291	0.91863	.717	.546	1	0.0017
Distal Skin Temperature	ALL (N=38)	RA_cos	Pearson correlation coeficient	-,325*	.061	-.281	.173	-.038	-.131	-.093	-.101	-0.1048	-.012	-.031	.004	.007	-.040	.146	.182	.207	0.27015	.073	.086	.293	.064	.196	-.196	-.023	.033	.046	-.170	0.02272	.057	-.099		
			Sig. (bilateral)	.046	.717	.088	.298	.822	.433	.578	.547	0.53126	.941	.851	.981	.966	.813	.382	.275	.213	.101	.663	.606	.074	.702	.238	.238	.894	.845	.789	.308	0.8923	.733	.553	1	0.0017
Distal Skin Temperature	ALL (N=38)	RA_np	Pearson correlation coeficient	-,407*	-.001	-,374*	.134	-.082	-.185	-.146	-.189	-0.1658	-.044	-.107	-.071	-.082	-.127	.192	.269	.290	,339*	.146	.172	.307	.106	.270	-.250	-.123	.042	-.030	-.247	-0.025	.021	-.169		
			Sig. (bilateral)	.011	.998	.021	.422	.626	.265	.383	.256	0.31987	.795	.524	.673	.625	.447	.249	.102	.078	.037	.382	.303	.061	.527	.101	.129	.467	.803	.860	.135	0.88156	.901	.310	3	0.0147
Distal Skin Temperature	ALL (N=38)	IV	Pearson correlation coeficient	-.029	.140	.000	.125	.078	.205	.155	.077	0.05207	.137	.020	-.140	.108	.061	-.281	.040	-.067	-0.0824	-.038	.004	-.303	-.084	-.116	-.018	-.016	.187	-.011	-.070	0.04309	.121	.067		
			Sig. (bilateral)	.862	.403	.998	.455	.640	.218	.353	.644	0.75618	.412	.907	.403	.518	.717	.087	.810	.688	.623	.820	.983	.064	.615	.487	.914	.925	.269	.950	.675	0.79727	.470	.689	0	
Distal Skin Temperature	ALL (N=38)	R	Pearson correlation coeficient	-.147	.067	-.102	.148	-.203	-.229	-.229	-.030	-0.1116	.183	-.005	.260	.006	.033	-.064	-.003	.036	0.00814	-.092	.027	.252	.069	.030	-.145	.024	.012	-.043	-.032	-0.1591	.207	-.049		
			Sig. (bilateral)	.379	.691	.543	.375	.222	.167	.166	.858	0.50479	.271	.974	.114	.970	.845	.701	.987	.831	.961	.582	.872	.127	.682	.859	.385	.888	.944	.801	.847	0.33995	.213	.770	0	
Distal Skin Temperature	ALL (N=38)	PV	Pearson correlation coeficient	-.140	-.019	-.156	.045	-.112	-.143	-.136	-.066	-0.0926	.110	-.054	.119	-.005	-.016	.090	.108	.155	0.14821	.026	.145	,324*	.060	.156	-.208	-.091	-.173	-.155	-.241	-0.018	.111	-.186		
			Sig. (bilateral)	.403	.910	.349	.787	.502	.393	.415	.693	0.5805	.511	.750	.478	.978	.924	.593	.519	.353	.375	.876	.386	.047	.722	.350	.210	.594	.305	.358	.144	0.91447	.508	.264	1	0.0017
Distal Skin Temperature	ALL (N=38)	M5	Pearson correlation coeficient	.130	-,335*	-.025	-,399*	.146	.163	.164	-.027	-0.041	.014	-.027	-.167	-.081	-.058	.202	.221	.297	0.16679	.087	.279	.186	-.198	.207	.002	-.278	-.305	-,393*	-,442**	0.09134	-.034	-.221		
			Sig. (bilateral)	.438	.040	.883	.013	.380	.329	.325	.874	0.80707	.931	.873	.317	.629	.728	.223	.181	.070	.317	.602	.090	.263	.234	.212	.990	.096	.067	.016	.005	0.58549	.842	.183	4	0.02
Distal Skin Temperature	ALL (N=38)	L10	Pearson correlation coeficient	,460**	-.114	,370*	-.274	.136	.251	.210	.185	0.15693	.042	.102	.010	.064	.112	-.121	-.192	-.185	-0.2758	-.117	-.077	-.236	-.176	-.197	.247	.021	-.160	-.116	.087	0.06883	-.028	.092		
			Sig. (bilateral)	.004	.495	.022	.096	.416	.128	.207	.265	0.34676	.804	.541	.952	.700	.503	.470	.247	.267	.094	.485	.644	.153	.290	.236	.135	.903	.344	.493	.603	0.68136	.866	.581	2	0.0081
Distal Skin Temperature	ALL (N=38)	P1_T	Pearson correlation coeficient	.005	.138	.028	.163	.093	.046	.072	.115	0.04124	.091	.119	.173	.203	.157	-.021	-.075	-.005	0.05471	-.142	-.059	.250	-.111	-.012	-.005	.139	-.164	.044	-.060	0.15688	.190	.017		
			Sig. (bilateral)	.978	.407	.869	.329	.577	.785	.669	.492	0.8058	.587	.476	.300	.223	.347	.902	.656	.977	.744	.394	.725	.131	.507	.945	.975	.411	.332	.797	.719	0.34689	.253	.920	0	
Distal Skin Temperature	ALL (N=38)	P2_T	Pearson correlation coeficient	-.310	-.279	-,385*	-.188	-.319	-,371*	-,367*	-,336*	-0.2547	.017	-,343*	-.095	-,380*	-,330*	.230	,334*	,336*	0.28384	.277	.296	.194	.285	,323*	-.300	-,340*	-.076	-,395*	-,343*	-0.2362	-.160	-,368*		
			Sig. (bilateral)	.058	.089	.017	.258	.051	.022	.023	.039	0.12275	.922	.035	.568	.018	.043	.164	.040	.039	.084	.093	.071	.243	.083	.048	.068	.039	.654	.015	.035	0.15332	.337	.023	14	0.0517
Distal Skin Temperature	ALL (N=38)	P3_T	Pearson correlation coeficient	.139	,336*	.226	.286	-.039	.124	.051	.277	0.07037	,365*	.242	,365*	.167	.276	-.301	-.254	-.264	-0.2849	-.299	-.036	.046	-.021	-.216	-.098	.069	-.012	.222	.204	-0.006	,324*	.153		
			Sig. (bilateral)	.404	.039	.172	.082	.815	.459	.759	.093	0.67462	.024	.144	.024	.317	.093	.066	.123	.110	.083	.068	.831	.783	.902	.193	.556	.684	.942	.186	.220	0.97144	.047	.358	4	0.02
