# Supplementary figures and images for: Circadian rhythm abnormalities and autonomic dysfunction in patients with Chronic Fatigue Syndrome/Myalgic Encephalomyelitis
Source: PLoS One. 2018 Jun 6;13(6):e0198106. doi: 10.1371/journal.pone.0198106 (PMC5991397; doi:10.1371/journal.pone.0198106)

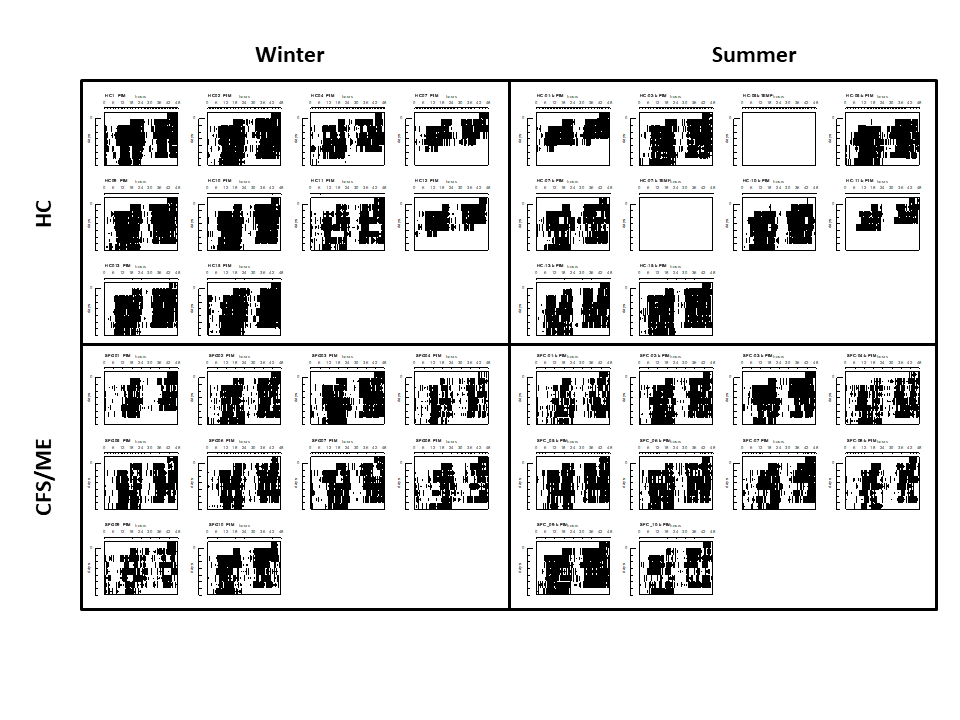

Supplement: S1 Fig — (TIF) [file pone.0198106.s003.tif]
